# Supplementary material for: Pharmacologic interventions for postoperative nausea and vomiting after thyroidectomy: A systematic review and network meta-analysis
Source: PLoS One. 2021 Jan 11;16(1):e0243865. doi: 10.1371/journal.pone.0243865 (PMC7799806; doi:10.1371/journal.pone.0243865)
Supplement: S1 Search Term — (DOCX) [file pone.0243865.s002.docx]

**Search Term**

Medline

1. randomized controlled trial.pt
2. randomized controlled trial$.mp
3. controlled clinical trial.pt
4. controlled clinical trial$.mp
5. random allocation.mp
6. exp double-blind method/
7. double-blind.mp
8. exp single-blind method/
9. single-blind.mp
10. or/1-9
11. clinical trial.pt
12. clinical trial$.mp
13. exp clinical trial/
14. (clin$ adj25 trial$).mp
15. ((singl$ or doubl$ or tripl$ or trebl$) adj25 (blind$ or mask$)).mp
16. random$.mp
17. exp research design/
18. research design.mp
19. or/11-18
20. 10 or 19
21. Case report.tw.
22. Letter.pt.
23. Historical article.pt.
24. Review.pt.
25. or/21-24
26. 20 not 25
27. exp postoperative nausea and vomiting/
28. vomit* OR nausea* OR PONV.mp
29. (postoperative adj6 nausea adj6 vomiting).mp
30. exp antiemetics/
31. exp serotonin antagonists/
32. 5-HT_3_ antagonist$.ti,ab.
33. 5-HT_3_ blocker$.ti,ab.
34. Ondansetron/ or granisetron/ or palonosetron/ or ramosetron/ or tropisetron/ or dolasetron/
35. Exp dopamine antagonists/
36. Metoclopramide/
37. (Metoclopramide or metaclopramide or metaclopromide or cerucal or maxolon or primperan or raglan or rimetin).ti,ab.
38. .Domperidone/
39. (Domperidone or domperidon or apo-domperidone or domidon or domperidon-teva or gastrocure or motilium or nauzelin or novo-domperidone or nu-domperidone or pms-domperidone or peridys or ratio-domperidone).ti,ab.
40. Exp butyrophenone/
41. Exp Droperidol/
42. Droperidol/
43. (Droperidol or dehydrobenzperidol or droleptan or dehidrobenzperidol or inapsine or inapsine).ti,ab.
44. Haloperidol/
45. (Haloperidol or haldol or dozic or serenace).ti,ab.
46. Methotrimeprazine/
47. (Methotrimeprazine or levomeprazin or levopromazine or levomepromazine or tisercin or tizertsin or tizercine or levoprome or nozinan).ti,ab.
48. Beclomethasone/
49. (Beclomethasone or beclometasone or qvar or aerobec forte or beclazone or ecobec or filair or aerobec or nasobec aqueous or prolair or respocort or ventolair or vancenase or vanceril or aldecin or viarin or apo-beclomethasone or ascocortonyl or beclamet or beclocort or beclomet or beclorhinol or becloturmant or sanasthmax or beclovent or beconase or propaderm or sanasthmyl or becodisks or becotide or becloforte or bronchocort or junik or asmabec clickhaler or beclazone or clenil modulate).ti,ab.
50. Betamethasone/
51. (Betamethasone or betadexamethasone or flubenisolone or celeston or celestona or celestone or cellestoderm or Betnelan or betnesol).ti,ab.
52. betamethasone 17-valerate/
53. (betamethasone 17-valerate or flubenisolonvalerate or betnovate or Beta-val or betaderm or betatrex or dermabet or luxiq or valisone or valnac or betacap or betamethasone valerate or bettamousse or diprosone).ti,ab.
54. Budesonide/
55. (Budesonide or horacort or pulmicort or rhinocort or novolizer or entocort).ti,ab.
56. Dexamethasone/
57. (Dexamethasone or hexadecadrol or methylfluorprednisolone or dexpak or maxidex or decaject or decameth or decaspray or dexasone or hexadrol or millicorten or oradexon or aeroseb-dex or decaderm dexamethasone or decadron or decadron or mymethasone).ti,ab.
58. dexamethasone isonicotinate/
59. (dexamethasone isonicotinate or auxison).ti,ab.
60. flumethasone/
61. (Flumethasone or fluorodexamethasone or locorten).ti,ab.
62. Fluorometholone/
63. (Fluorometholone or cortisdin or flucon or fluoro-ophtal or fml or pms-fluorometholone or fluoropos or oxylone).ti,ab.
64. fluprednisolone/
65. (fluprednisolone or alphadrol).ti,ab.
66. Flurandrenolone/
67. (Flurandrenolone or flurandrenolide or cordran or haelan or fludroxycortide).ti,ab.
68. melengestrol acetate/
69. (melengestrol acetate or melengestrol).ti,ab.
70. Methylprednisolone/
71. (Methylprednisolone or metipred or medrol or urbason or medrone or solu-medrone or depo-medrone).ti,ab.
72. methylprednisolone hemisuccinate/
73. (a-methapred or solu-medrol or solumedrol or urbason-soluble or urbasonsoluble).ti,ab.
74. Prednisolone/
75. (Prednisolone or di-adreson-f or diadresonf or predate or predonine or cortalone or delta-cortef or fernisolone-p or meti-derm or prelone or sterane).ti,ab.
76. Prednisone/
77. (Prednisone or dehydrocortisone or delta-cortisone or prednison galen or prednison hexal or pronisone or rectodelt or apo-prednisone or cortancyl or panafcort or dacortin or deltasone or prednidib or predni tablinen or panasol or orasone or meticorten or liquid pred or kortancyl or enkortolon or encortone or encorton or prednison acsis or predniment or decortisyl or cutason or cortan or winpred or ultracorten or sone or sterapred or delta-dome or fernisone or paracort or predincen-m or servisone).ti,ab.
78. Cyclizine/
79. (Cyclizine or marezine or valoid).ti,ab.
80. exp Benzodiazepines/
81. (Alprazolam or Alprazolan or alprox or esparon or apo-alpraz or apoalpraz or cassadan or d-65mt or d65mt or kalma or novo-alprazol or novoalprazol or nu-alpraz or nualpraz or ralozam or u-31,889 or u31,889 or xanax or tafil or trankimazin or Niravam).ti,ab.
82. (Clonazepam or antelepsin or rivotril or ro 5-4023 or ro 54023 or klonopin).ti,ab.
83. (Diazepam or apaurin or diazemuls or faustan or relanium or seduxen or sibazon or stesolid or valium or rimapam or tensium or dialar or valclair or diastat or dizac or q-pam or valrelease).ti,ab.
84. (Flumazenil or flumazepil or romazicon or anexate or lanexat or ro 15-1788 or ro 151788 or Anexate).ti,ab.
85. (Lorazepam or apo-lorazepam or apolorazepam or ativan or orfidal or temesta or donix or duralozam or durazolam or idalprem or laubeel or lorazep von ct or novo-lorazem or novolorazem or nu-loraz or nuloraz or sedicepan or sinestron or somagerol or tolid or wy-4036 or wy4036 or loraz).ti,ab.
86. (Oxazepam or adumbran or serax or tazepam).ti,ab.
87. (Pirenzepine or pirenzepin or pyrenzepine or ulcoprotect or ulgescum or gastrotsepin or piren-basan or pirenzepin-ratiopharm or gastrozepin).ti,ab.
88. (Chlordiazepoxide or methaminodiazepoxide or chlozepid or elenium or librium or a-poxide or chlordiazachel or librelease or libritabs or lygen).ti,ab.
89. (Chlorazepate or tranxene or tranxilium).ti,ab.
90. (Estazolam or nuctalon or prosom or tasedan).ti,ab.
91. (Midazolam or dormicum or ro 21-3981 or ro 213981 or versed or hypnovel).ti,ab.
92. (Triazolam or apo-triazo or gen-triazolam or halcyon or halcion or trilam).ti,ab.
93. aprepitant/
94. Or/27-93
95. 26 and 94
96. Thyroidectomy/
97. thyroidectom*.tw.
98. or/96-97
99. 95 and 98

EMBASE

1. randomi?ed controlled trial$.mp.
2. 'controlled clinical trial'/exp
3. controlled AND clinical AND trials
4. controlled clinical trial$.mp.
5. 'randomization'/exp
6. 'random allocation'/exp
7. random allocation.mp.
8. double-blind.mp.
9. single-blind.mp.
10. #1 OR #2 OR #3 OR #4 OR #5 OR #6 OR #7 OR #8 OR #9
11. 'clinical trial (topic)'/exp
12. clinical AND trial$.mp.
13. random$.mp.
14. rct
15. #11 OR #12 OR #13 OR #14
16. #10 OR #15
17. 'case study'/exp
18. 'case report'/exp
19. 'abstract report'/exp
20. 'letter'/exp
21. #17 OR #18 OR #19 OR #20
22. #16 NOT #21
23. 'postoperative nausea and vomiting'/exp
24. 'postoperative nausea'/exp
25. 'postoperative vomiting'/exp
26. Nausea or vomiting or PONV.mp.
27. ‘antiemetic agent’/exp
28. ‘serotonin antagonists’/exp
29. Ondansetron or granisetron or palonosetron or ramosetron or tropisetron or dolasetron
30. ‘dopamine receptor blocking agent’/exp
31. Metoclopramide
32. Metoclopramide or metaclopramide or metaclopromide or cerucal or maxolon or primperan or raglan or rimetin
33. Domperidone
34. (Domperidone or domperidon or apo-domperidone or domidon or domperidon-teva or gastrocure or motilium or nauzelin or novo-domperidone or nu-domperidone or pms-domperidone or peridys or ratio-domperidone)
35. ‘butyrophenone derivative’/exp
36. ‘Droperidol’/exp
37. (Droperidol or dehydrobenzperidol or droleptan or dehidrobenzperidol or inapsine or inapsine).
38. ‘Haloperidol’/exp
39. (Haloperidol or haldol or dozic or serenace)
40. (Methotrimeprazine or levomeprazin or levopromazine or levomepromazine or tisercin or tizertsin or tizercine or levoprome or nozinan)
41. ‘steroid’/exp
42. Beclometasone
43. (Beclomethasone or beclometasone or qvar or aerobec forte or beclazone or ecobec or filair or aerobec or nasobec aqueous or prolair or respocort or ventolair or vancenase or vanceril or aldecin or viarin or apo-beclomethasone or ascocortonyl or beclamet or beclocort or beclomet or beclorhinol or becloturmant or sanasthmax or beclovent or beconase or propaderm or sanasthmyl or becodisks or becotide or becloforte or bronchocort or junik or asmabec clickhaler or beclazone or clenil modulate)
44. Betamethasone
45. (Betamethasone or betadexamethasone or flubenisolone or celeston or celestona or celestone or cellestoderm or Betnelan or betnesol)
46. Betamethasone Valerate
47. (betamethasone 17-valerate or flubenisolonvalerate or betnovate or Beta-val or betaderm or betatrex or dermabet or luxiq or valisone or valnac or betacap or betamethasone valerate or bettamousse or diprosone)
48. Budesonide
49. (Budesonide or horacort or pulmicort or rhinocort or novolizer or entocort)
50. Dexamethasone
51. (Dexamethasone or hexadecadrol or methylfluorprednisolone or dexpak or maxidex or decaject or decameth or decaspray or dexasone or hexadrol or millicorten or oradexon or aeroseb-dex or decaderm dexamethasone or decadron or decadron or mymethasone)
52. Dexamethasone Isonicotinate
53. (dexamethasone isonicotinate or auxison)
54. Methylprednisolone
55. (Methylprednisolone or metipred or medrol or urbason or medrone or solu-medrone or depo-medrone)
56. Methylprednisolone Sodium Succinate
57. (a-methapred or solu-medrol or solumedrol or urbason-soluble or urbasonsoluble).ti,ab.
58. Prednisolone
59. (Prednisolone or di-adreson-f or diadresonf or predate or predonine or cortalone or delta-cortef or fernisolone-p or meti-derm or prelone or sterane).
60. Prednisone
61. (Prednisone or dehydrocortisone or delta-cortisone or prednison galen or prednison hexal or pronisone or rectodelt or apo-prednisone or cortancyl or panafcort or dacortin or deltasone or prednidib or predni tablinen or panasol or orasone or meticorten or liquid pred or kortancyl or enkortolon or encortone or encorton or prednison acsis or predniment or decortisyl or cutason or cortan or winpred or ultracorten or sone or sterapred or delta-dome or fernisone or paracort or predincen-m or servisone).
62. Cyclizine
63. (Cyclizine or marezine or valoid)
64. Chlorpromazine
65. (Chlorpromazine or propaphenin or aminazine or chlordelazine or contomin or largactil or fenactil or chlorazine or thorazine or thorazine).ti,ab.
66. Benzodiazepine or Alprazolam or Anthramycin or Bromazepam or Clonazepam or Devazepide or Diazepam or Nordazepam or Flumazenil or Lorazepam or Flunitrazepam or Flurazepam or Nitrazepam or Oxazepam or Pirenzepine or Prazepam or Temazepam or Chlordiazepoxide or Clorazepate or Estazolam or Medazepam or Midazolam or Triazolam
67. (Alprazolam or Alprazolan or alprox or esparon or apo-alpraz or apoalpraz or cassadan or d-65mt or d65mt or kalma or novo-alprazol or novoalprazol or nu-alpraz or nualpraz or ralozam or u-31,889 or u31,889 or xanax or tafil or trankimazin or Niravam).
68. (Bromazepam or anxyrex or apo-bromazepam or bromalich or bromaz or bromazanil or bromazep or lexotan or lexomil or lexotanil or lexatin or ro 5-3350 or ro 53350 or durazanil or gen-bromazepam).
69. (Clonazepam or antelepsin or rivotril or ro 5-4023 or ro 54023 or klonopin).
70. (Diazepam or apaurin or diazemuls or faustan or relanium or seduxen or sibazon or stesolid or valium or rimapam or tensium or dialar or valclair or diastat or dizac or q-pam or valrelease).
71. (Nordazepam or demethyldiazepam or desmethyldiazepam or deoxydemoxepam or nordiazepam or norprazepam or dealkylprazepam or calmday or nordaz or tranxilium n or vegesan).
72. (Flumazenil or flumazepil or romazicon or anexate or lanexat or ro 15-1788 or ro 151788 or Anexate).
73. (Lorazepam or apo-lorazepam or apolorazepam or ativan or orfidal or temesta or donix or duralozam or durazolam or idalprem or laubeel or lorazep von ct or novo-lorazem or novolorazem or nu-loraz or nuloraz or sedicepan or sinestron or somagerol or tolid or wy-4036 or wy4036 or loraz).
74. (Oxazepam or adumbran or serax or tazepam).
75. (Pirenzepine or pirenzepin or pyrenzepine or ulcoprotect or ulgescum or gastrotsepin or piren-basan or pirenzepin-ratiopharm or gastrozepin).
76. (Prazepam or centrax or demetrin or lysanxia or reapam).
77. (Chlordiazepoxide or methaminodiazepoxide or chlozepid or elenium or librium or a-poxide or chlordiazachel or librelease or libritabs or lygen).
78. (Chlorazepate or tranxene or tranxilium).
79. (Estazolam or nuctalon or prosom or tasedan).
80. (Medazepam or nobrium or ro 5-4556 or ro 54556 or rudotel or rusedal).
81. (Midazolam or dormicum or ro 21-3981 or ro 213981 or versed or hypnovel)..
82. (Triazolam or apo-triazo or gen-triazolam or halcyon or halcion or trilam).
83. ‘aprepitant’/exp
84. #23 OR #24 OR #25 OR #26 OR #27 OR #28 OR #29 OR #30 OR #31 OR #32 OR #33 OR #34 OR #35 OR #36 OR #37 OR #38 OR #39 OR #40 OR #41 OR #42 OR #43 OR #44 OR #45 OR #46 OR #47 OR #48 OR #49 OR #50 OR #51 OR #52 OR #53 OR #54 OR #55 OR #56 OR #57 OR #58 OR #59 OR #60 OR #61 OR #62 OR #63 OR #64 OR #65 OR #66 OR #67 OR #68 OR #69 OR #70 OR #71 OR #72 OR #73 OR #74 OR #75 OR #76 OR #77 OR #78 OR #79 OR #80 OR #81 OR #82 OR #83
85. #22 AND #84
86. ‘Thyroidectomy’/exp
87. ‘Subtotal thyroidectomy’/exp
88. thyroidectom*.tw.
89. #86 OR #87 OR #88
