# Supplementary material for: Pharmacologic interventions for postoperative nausea and vomiting after thyroidectomy: A systematic review and network meta-analysis
Source: PLoS One. 2021 Jan 11;16(1):e0243865. doi: 10.1371/journal.pone.0243865 (PMC7799806; doi:10.1371/journal.pone.0243865)
Supplement: S1 File — (DOCX) [file pone.0243865.s003.docx]

**S1 File_Manuscript figures**

**Result**

**Postoperative nausea and vomiting**


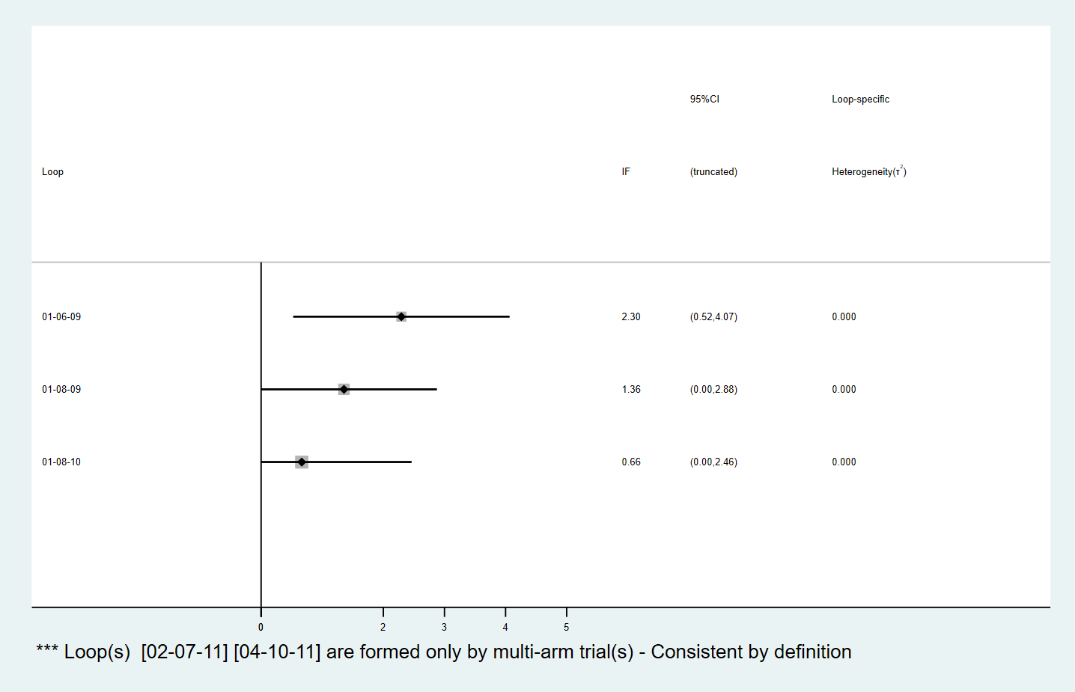


**S-Fig 4A. Inconsistency plot between the direct and indirect effect estimates for the same comparison for PONV.** Inconsistency factor (IF) as the absolute difference with 95% confidence interval (CI) between the direct and indirect estimates for each paired comparison is presented. IF values close to 0 indicate that the two sources are in agreement.


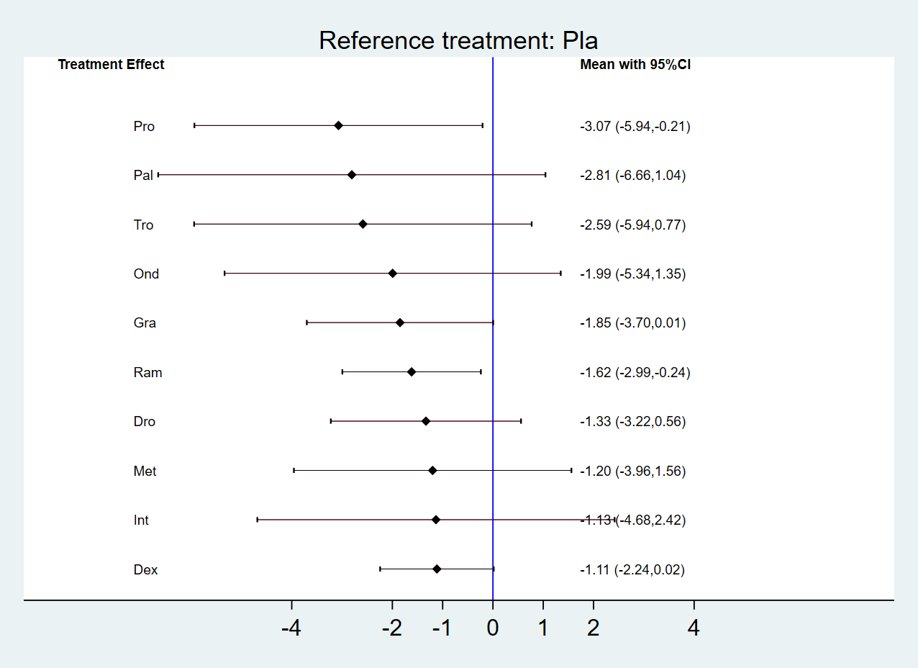


S-Fig 5A. Predictive interval plots between each management modality and placebo group for PONV. Diamond shape represents the mean summary effects. Black line represented the 95% CI, and red line represented the predictive interval (PrI). PrIs provide an interval that is expected to encompass the estimate of a future study.


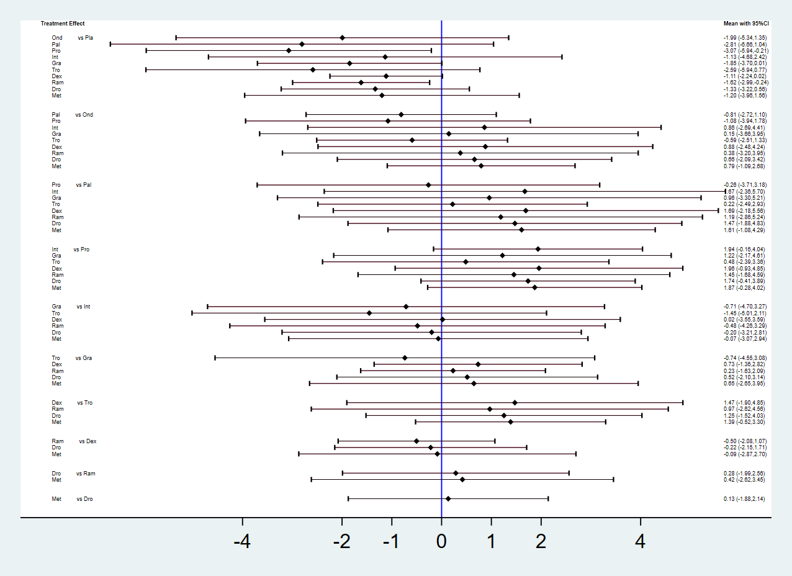


**S-Fig 6A. Predictive interval plots between each management modality for PONV.** Diamond shape represents the mean summary effects. Black line represented the 95% CI, and red line represented the predictive interval (PrI). PrIs provide an interval that is expected to encompass the estimate of a future study.


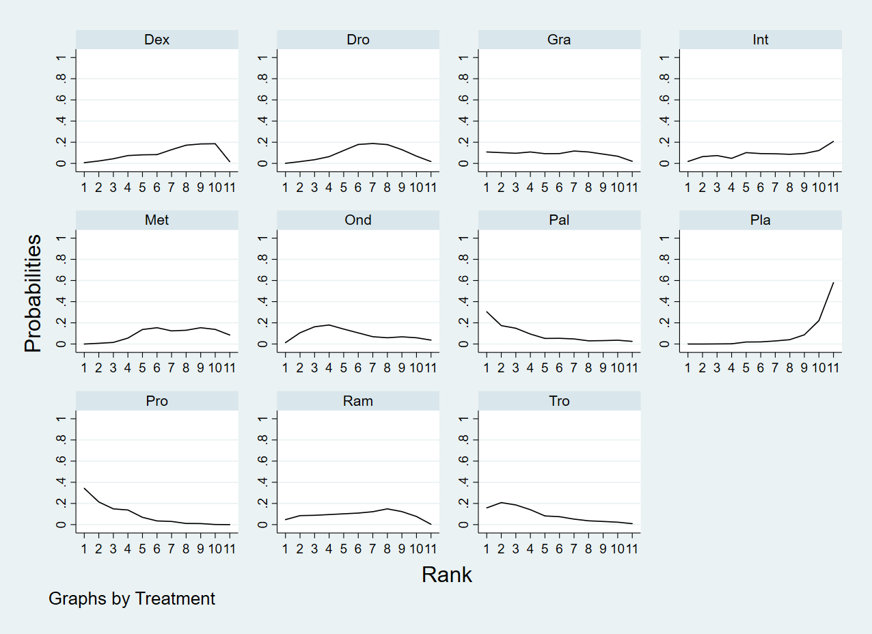


S-Fig 7A. Rankogram for PONV. Profiles indicate the probabilities for treatments to assume any of the possible ranks. It is the probability that a given treatment ranks first, second, third, and so on, among all of the treatments evaluated in the NMA.


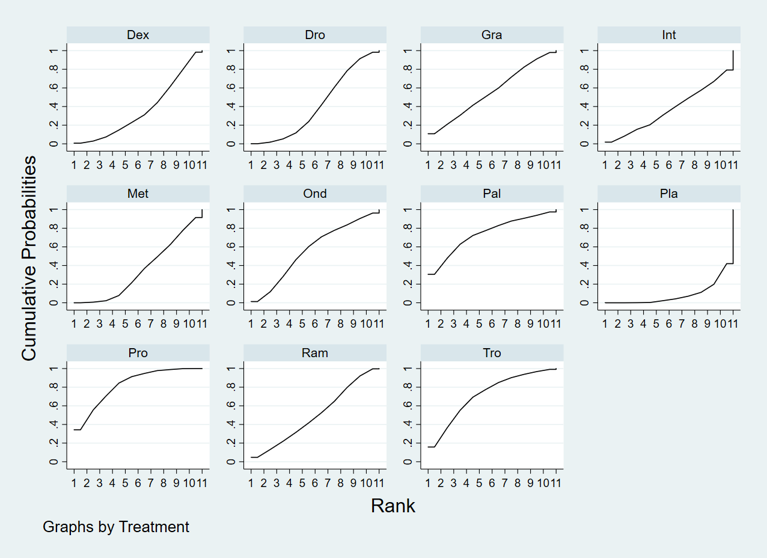


S-Fig 8A. Cumulative ranking curve plot for PONV. Profile indicates the sum of the probabilities from those ranked first, second, third, and so on. The surface under the cumulative ranking curve (SUCRA) value is regarded as an improved result for an individual’s intervention. When ranking treatments, the closer the SUCRA value is to 100%, the higher the treatment ranking is, relative to all other treatments.


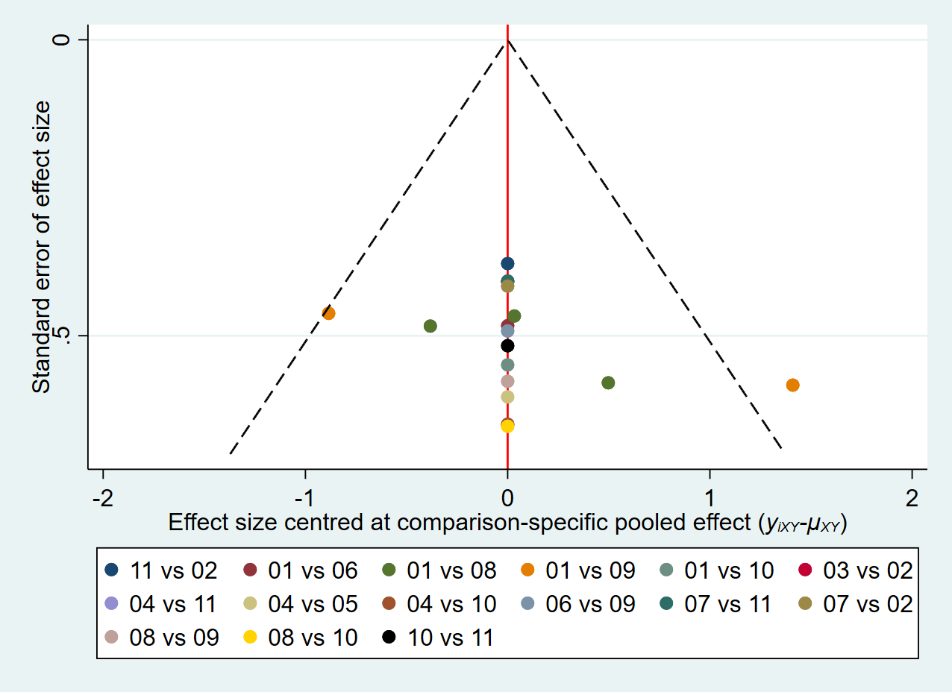


S-Fig 9A. Comparison-adjusted funnel plot for PONV.

**Postoperative nausea**


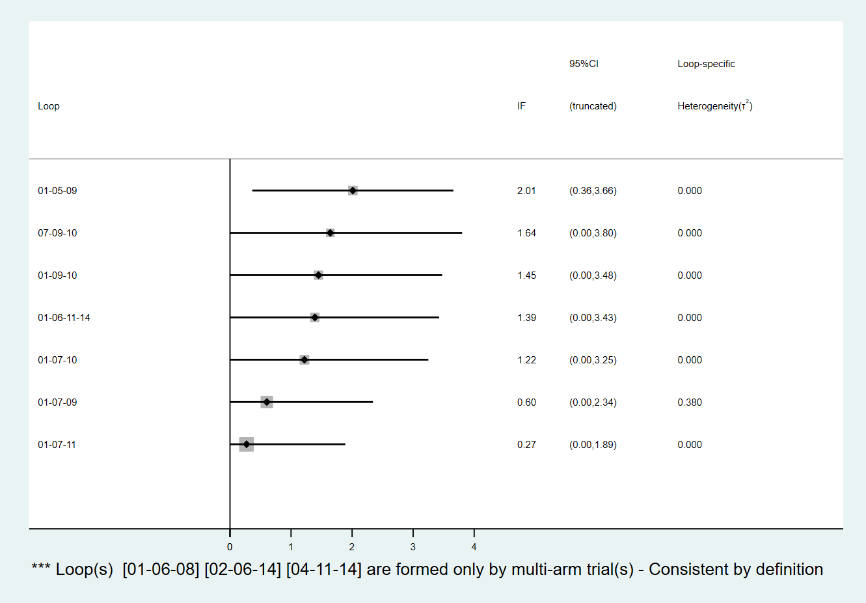


**S-Fig 4B. Inconsistency plot between the direct and indirect effect estimates for the same comparison for PON.** Inconsistency factor (IF) as the absolute difference with 95% confidence interval (CI) between the direct and indirect estimates for each paired comparison is presented. IF values close to 0 indicate that the two sources are in agreement.

**
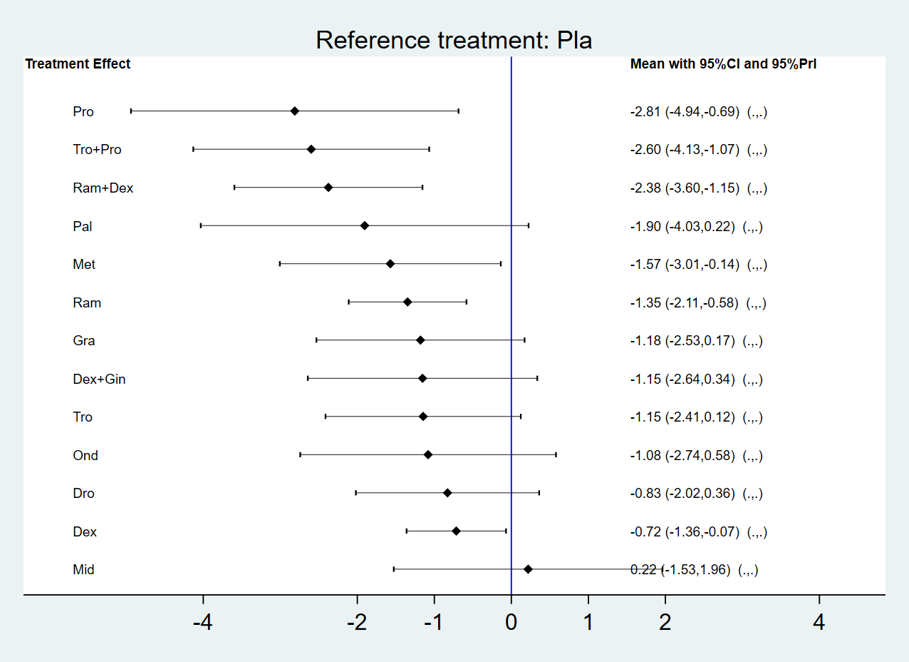
**

S-Fig 5B. Predictive interval plots between each management modality and placebo group for PON. Diamond shape represents the mean summary effects. Black line represented the 95% CI, and red line represented the predictive interval (PrI). PrIs provide an interval that is expected to encompass the estimate of a future study.


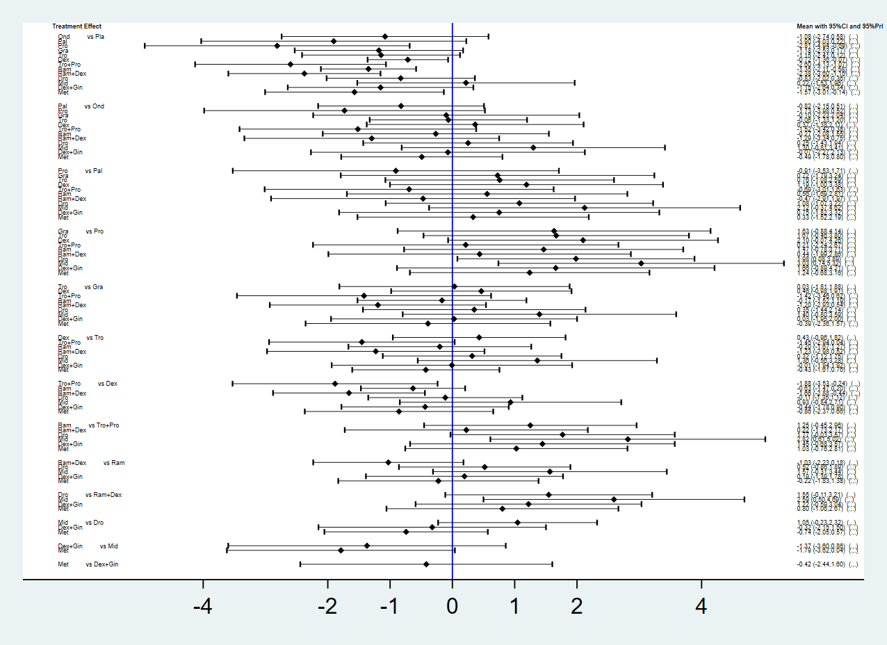


**S-Fig 6B. Predictive interval plots between each management modality for PON.** Diamond shape represents the mean summary effects. Black line represented the 95% CI, and red line represented the predictive interval (PrI). PrIs provide an interval that is expected to encompass the estimate of a future study.


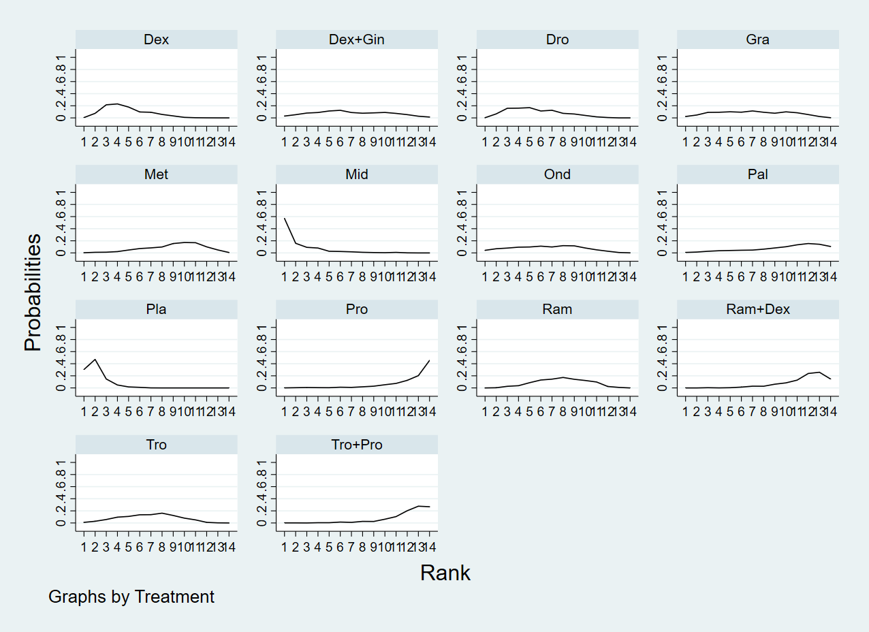


S-Fig 7B. Rankogram for PON. Profiles indicate the probabilities for treatments to assume any of the possible ranks. It is the probability that a given treatment ranks first, second, third, and so on, among all of the treatments evaluated in the NMA.


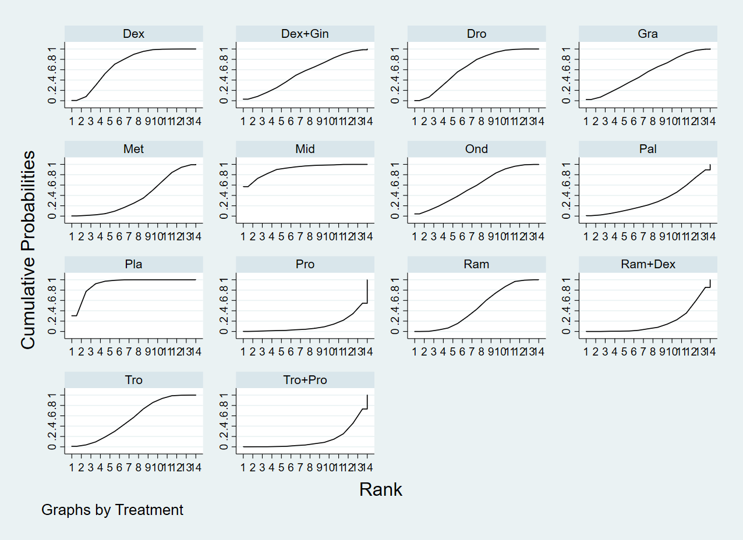


**S-Fig 8B. Cumulative ranking curve plot for PON.** Profile indicates the sum of the probabilities from those ranked first, second, third, and so on. The surface under the cumulative ranking curve (SUCRA) value is regarded as an improved result for an individual’s intervention. When ranking treatments, the closer the SUCRA value is to 100%, the higher the treatment ranking is, relative to all other treatments.


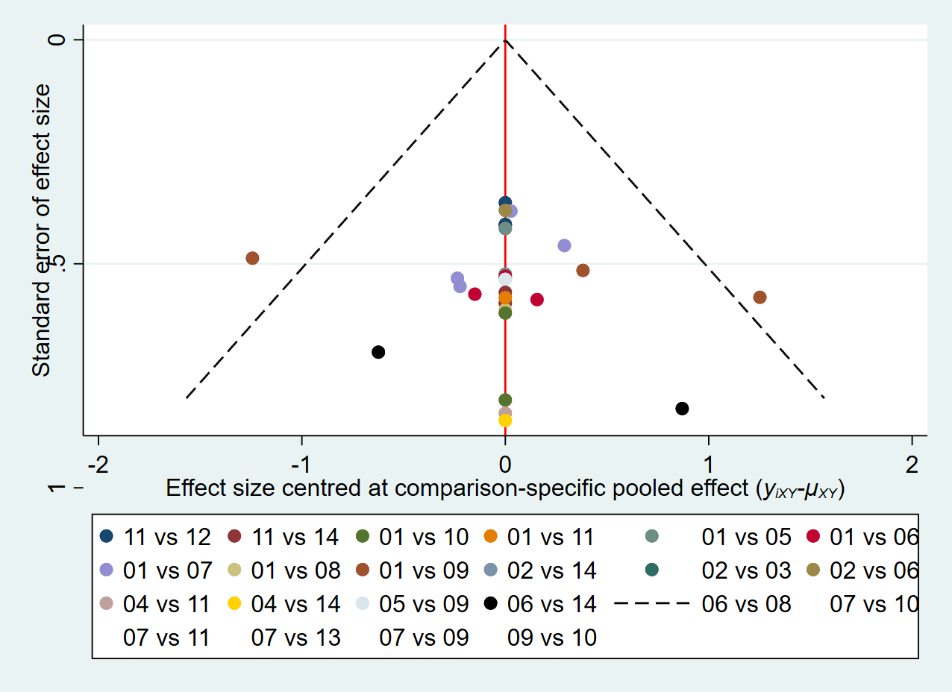


**S-Fig 9B. Comparison-adjusted funnel plot for PON.**

**Postoperative vomiting**


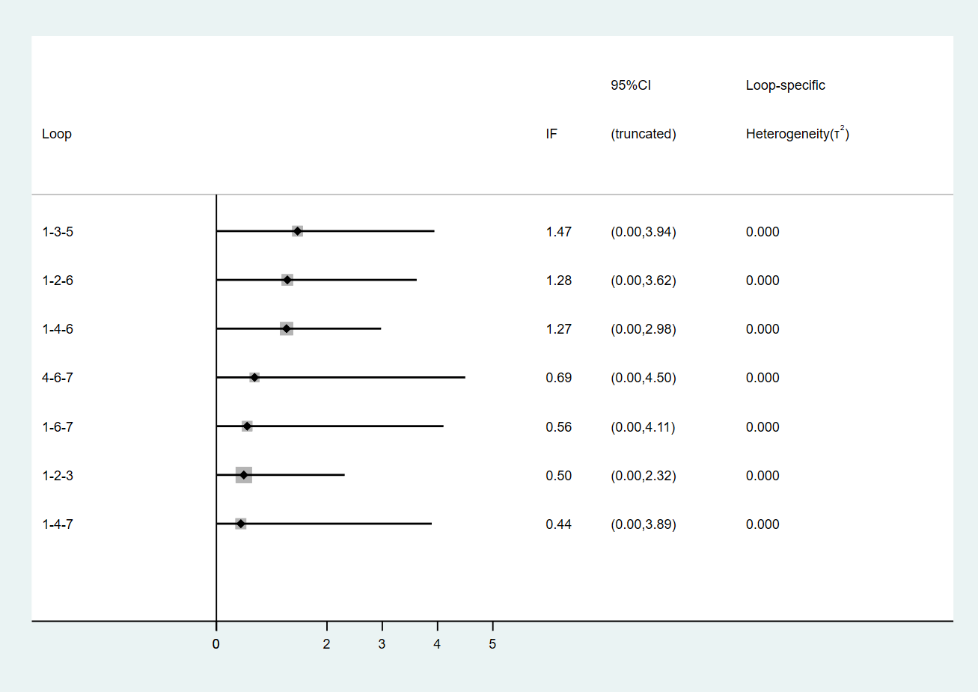


**S-Fig 4C. Inconsistency plot between the direct and indirect effect estimates for the same comparison for POV.** Inconsistency factor (IF) as the absolute difference with 95% confidence interval (CI) between the direct and indirect estimates for each paired comparison is presented. IF values close to 0 indicate that the two sources are in agreement.


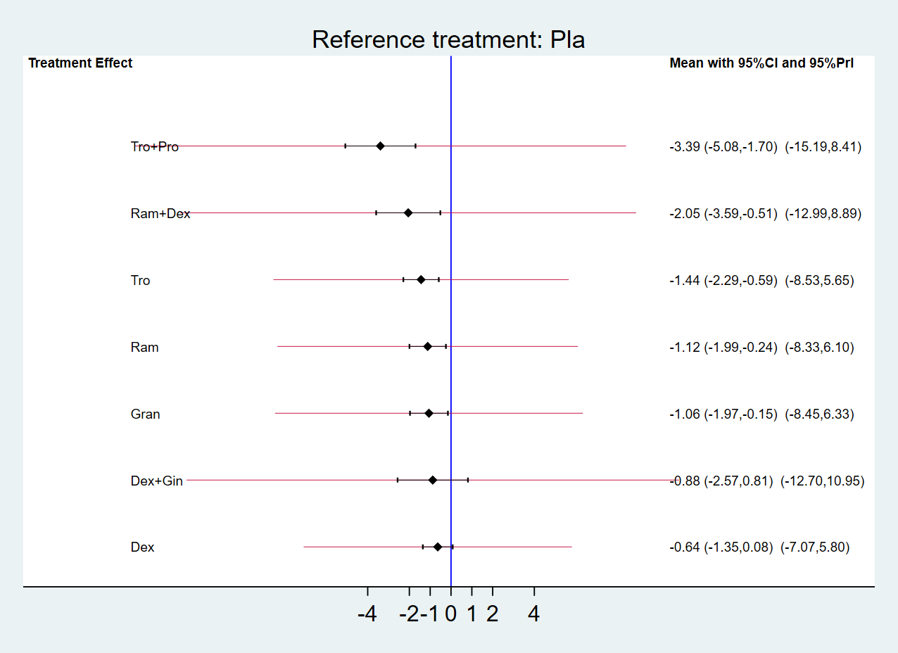


**S-Fig 5C. Predictive interval plots between each management modality and placebo group for POV.** Diamond shape represents the mean summary effects. Black line represented the 95% CI, and red line represented the predictive interval (PrI). PrIs provide an interval that is expected to encompass the estimate of a future study.


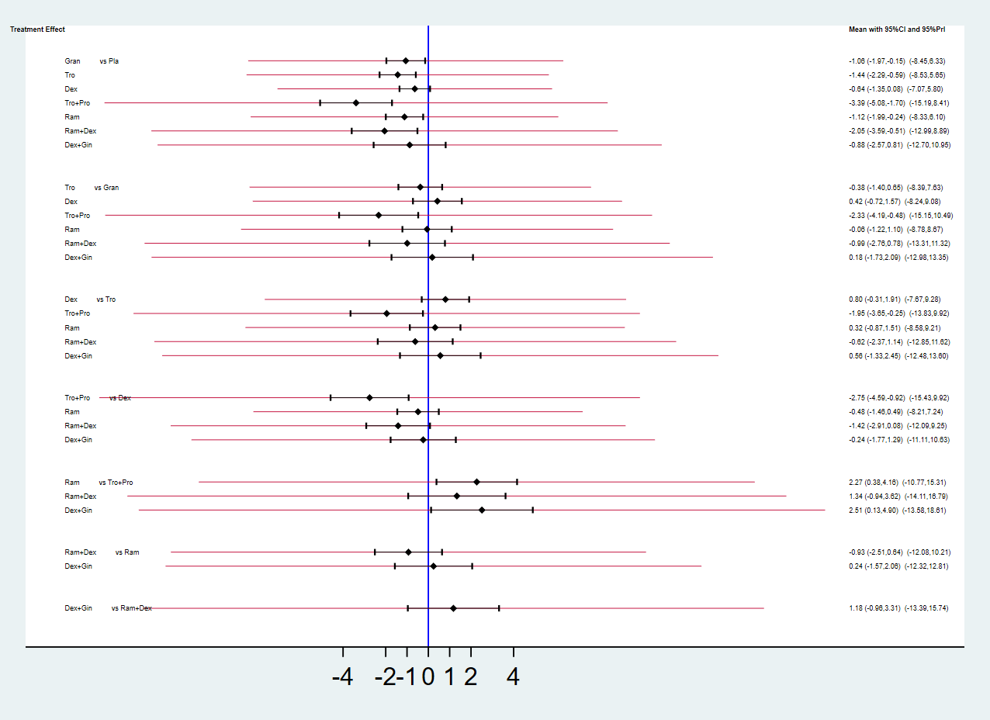


**S-Fig 6C. Predictive interval plots between each management modality for POV.** Diamond shape represents the mean summary effects. Black line represented the 95% CI, and red line represented the predictive interval (PrI). PrIs provide an interval that is expected to encompass the estimate of a future study.


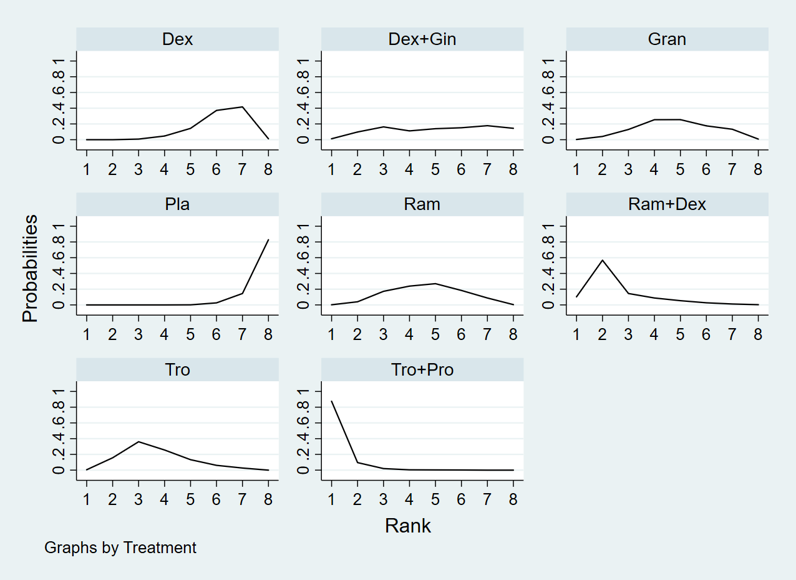


**S-Fig 7C. Rankogram for POV. Profiles indicate the probabilities for treatments to assume any of the possible ranks.** It is the probability that a given treatment ranks first, second, third, and so on, among all of the treatments evaluated in the NMA.


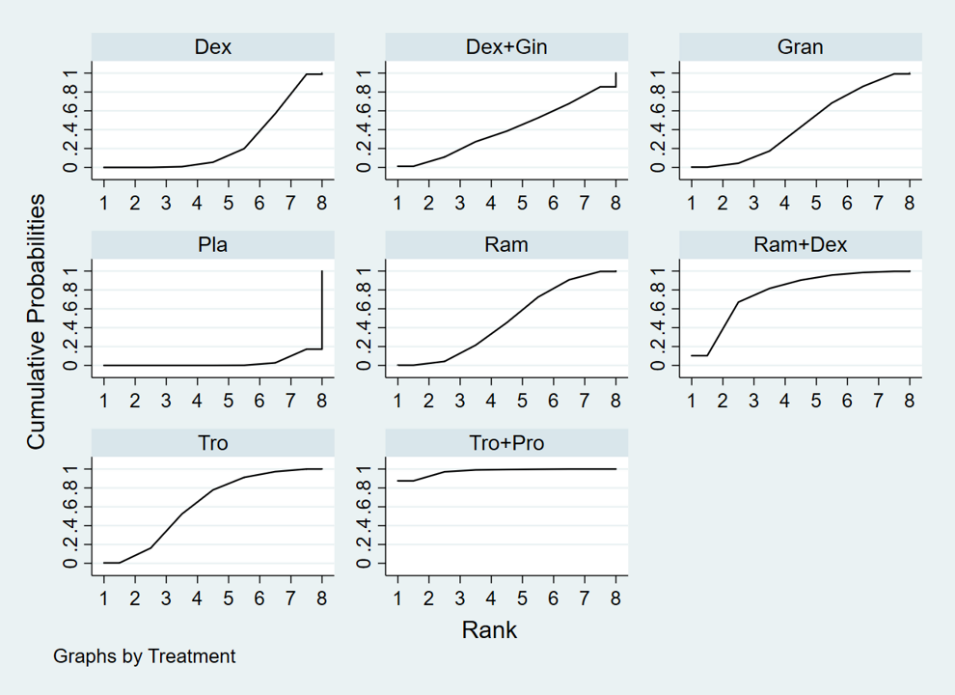


**S-Fig 8C. Cumulative ranking curve plot for POV.** Profile indicates the sum of the probabilities from those ranked first, second, third, and so on. The surface under the cumulative ranking curve (SUCRA) value is regarded as an improved result for an individual’s intervention. When ranking treatments, the closer the SUCRA value is to 100%, the higher the treatment ranking is, relative to all other treatments.


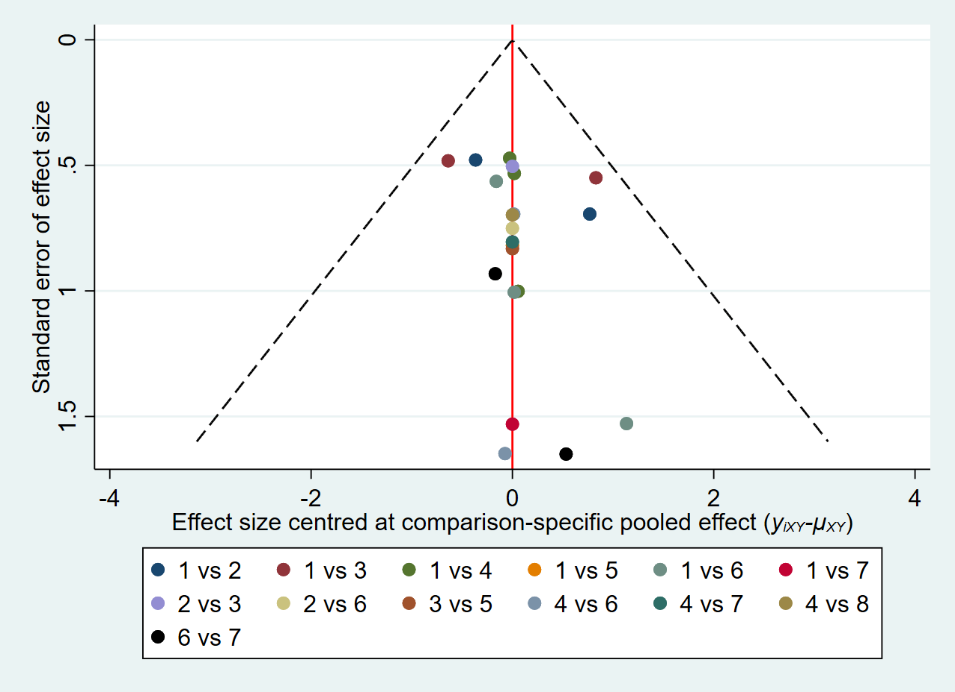


S-Fig 9C. Comparison-adjusted funnel plot for POV.

**Use of rescue antiemetics**


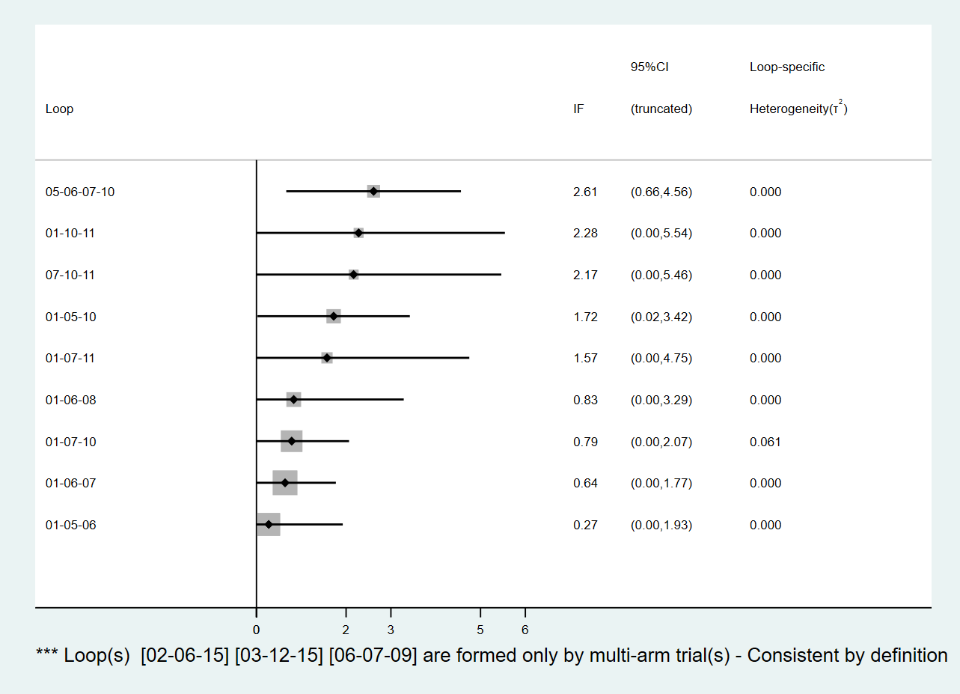


S-Fig 4D. Inconsistency plot between the direct and indirect effect estimates for the same comparison for use of rescue anti-emetics. Inconsistency factor (IF) as the absolute difference with 95% confidence interval (CI) between the direct and indirect estimates for each paired comparison is presented. IF values close to 0 indicate that the two sources are in agreement.


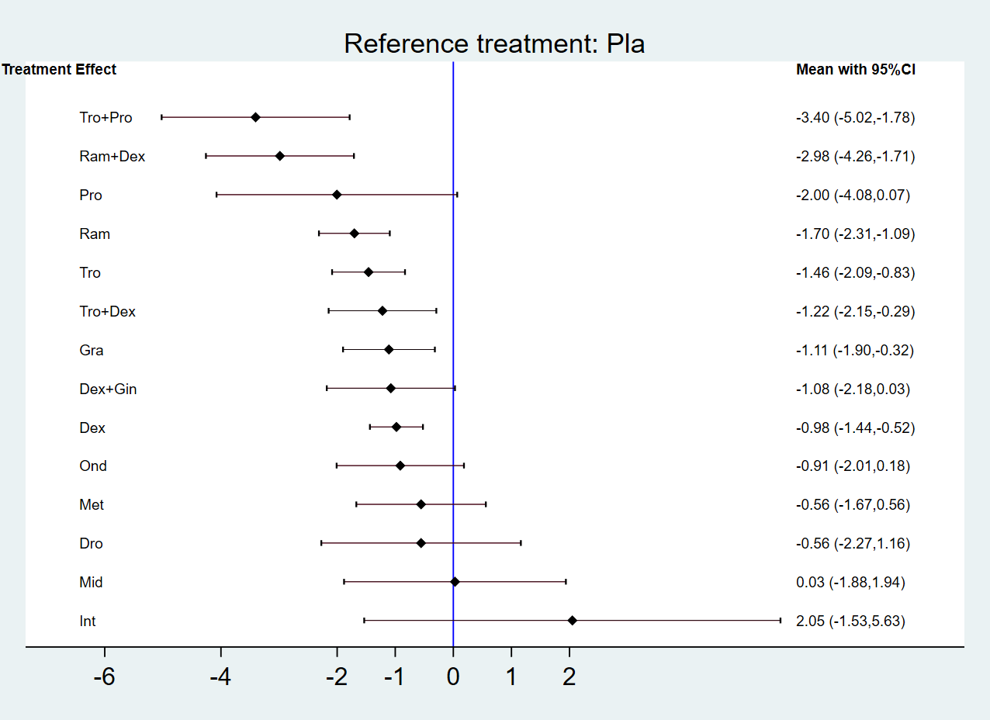


**S-Fig 5D. Predictive interval plots between each management modality and placebo group for use of rescue anti-emetics.** Diamond shape represents the mean summary effects. Black line represented the 95% CI, and red line represented the predictive interval (PrI). PrIs provide an interval that is expected to encompass the estimate of a future study.


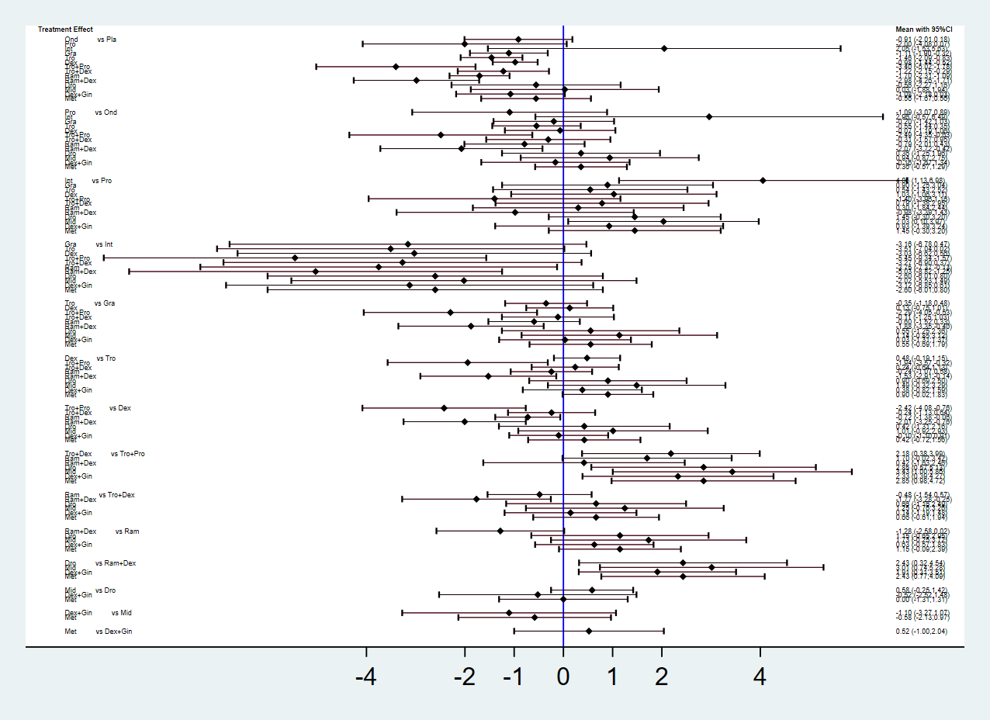


**S-Fig 6D.** **Predictive interval plots between each management modality for use of rescue anti-emetics.**

Diamond shape represents the mean summary effects. Black line represented the 95% CI, and red line represented the predictive interval (PrI). PrIs provide an interval that is expected to encompass the estimate of a future study.


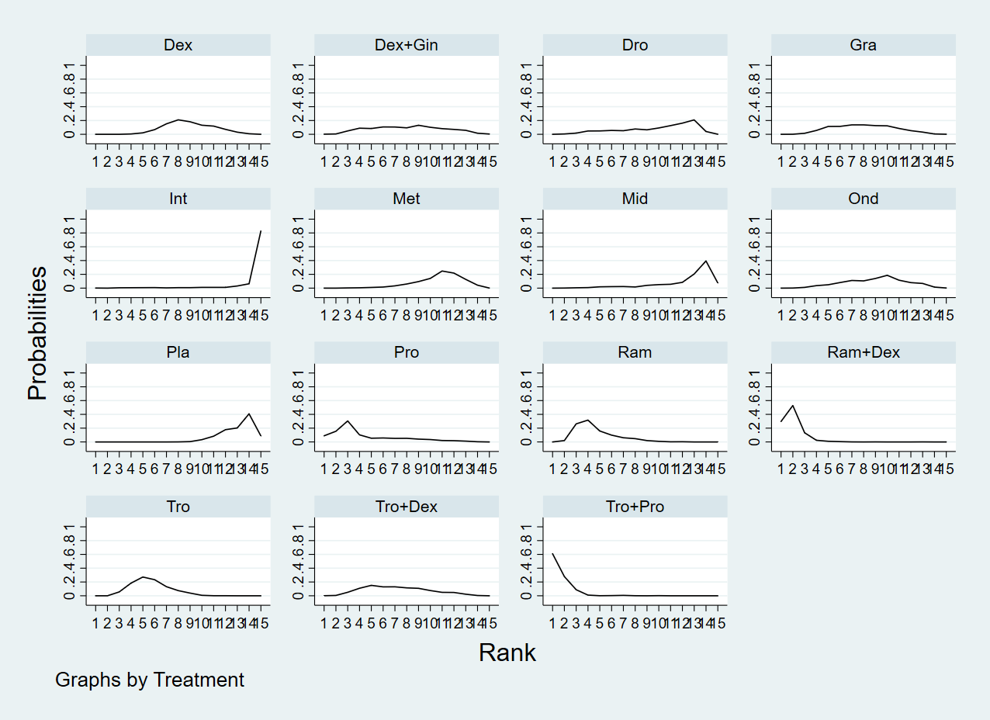


S-Fig 7D. Rankogram for use of rescue anti-emetics. Profiles indicate the probabilities for treatments to assume any of the possible ranks. It is the probability that a given treatment ranks first, second, third, and so on, among all of the treatments evaluated in the NMA.


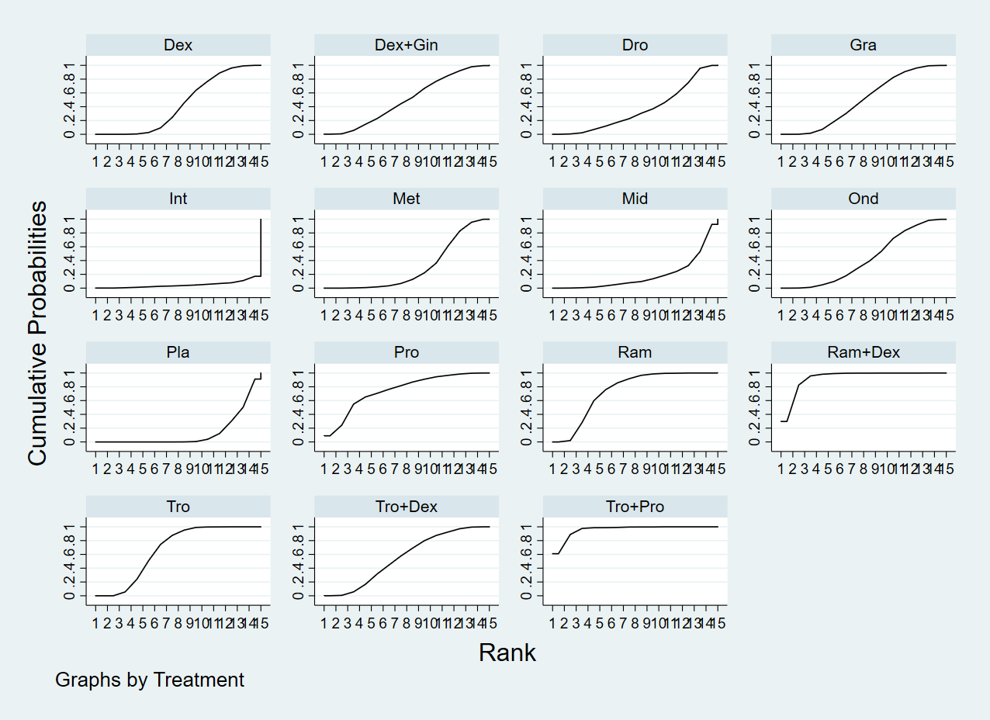


**S-Fig 8D.** **Cumulative ranking curve plot for use of rescue anti-emetics.** Profile indicates the sum of the probabilities from those ranked first, second, third, and so on. The surface under the cumulative ranking curve (SUCRA) value is regarded as an improved result for an individual’s intervention. When ranking treatments, the closer the SUCRA value is to 100%, the higher the treatment ranking is, relative to all other treatments.


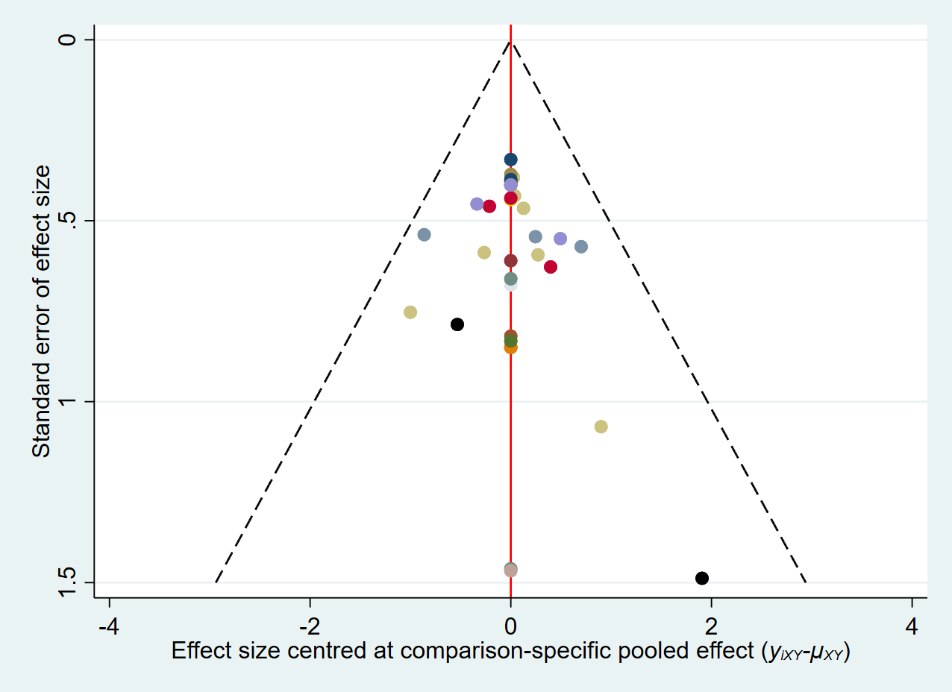


**S-Fig 9D. Comparison-adjusted funnel plot for use of rescue anti-emetics.**

**Complete response**


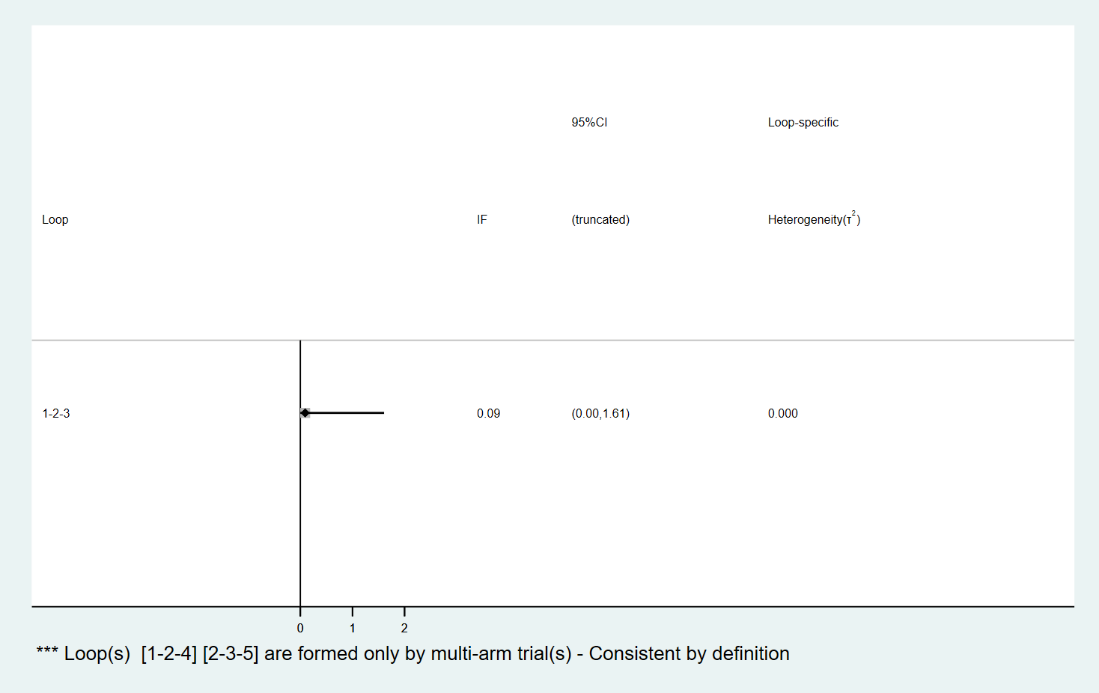


**S-Fig 4E. Inconsistency plot between the direct and indirect effect estimates for the same comparison for complete response.** Inconsistency factor (IF) as the absolute difference with 95% confidence interval (CI) between the direct and indirect estimates for each paired comparison is presented. IF values close to 0 indicate that the two sources are in agreement.


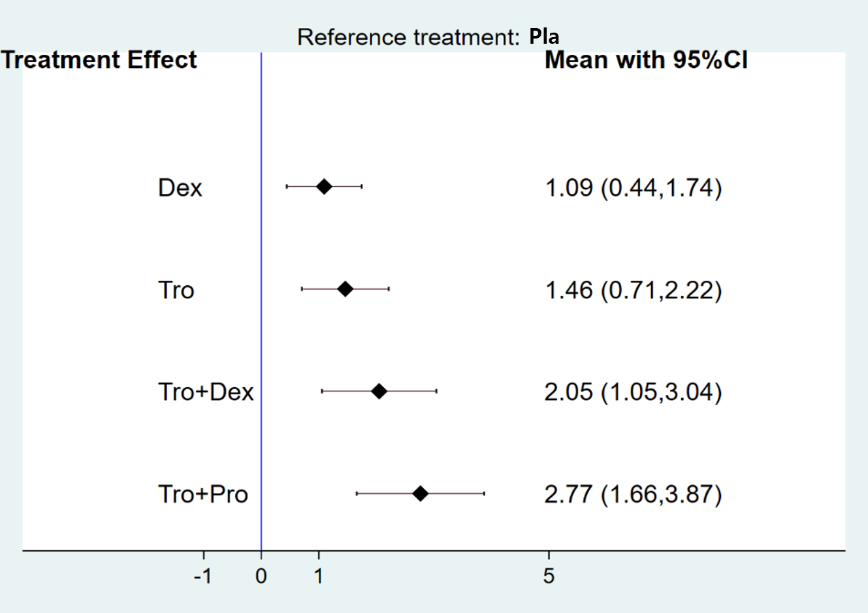


**S-Fig 5E.** **Predictive interval plots between each management modality and placebo group.** Diamond shape represents the mean summary effects. Black line represented the 95% CI, and red line represented the predictive interval (PrI). PrIs provide an interval that is expected to encompass the estimate of a future study.


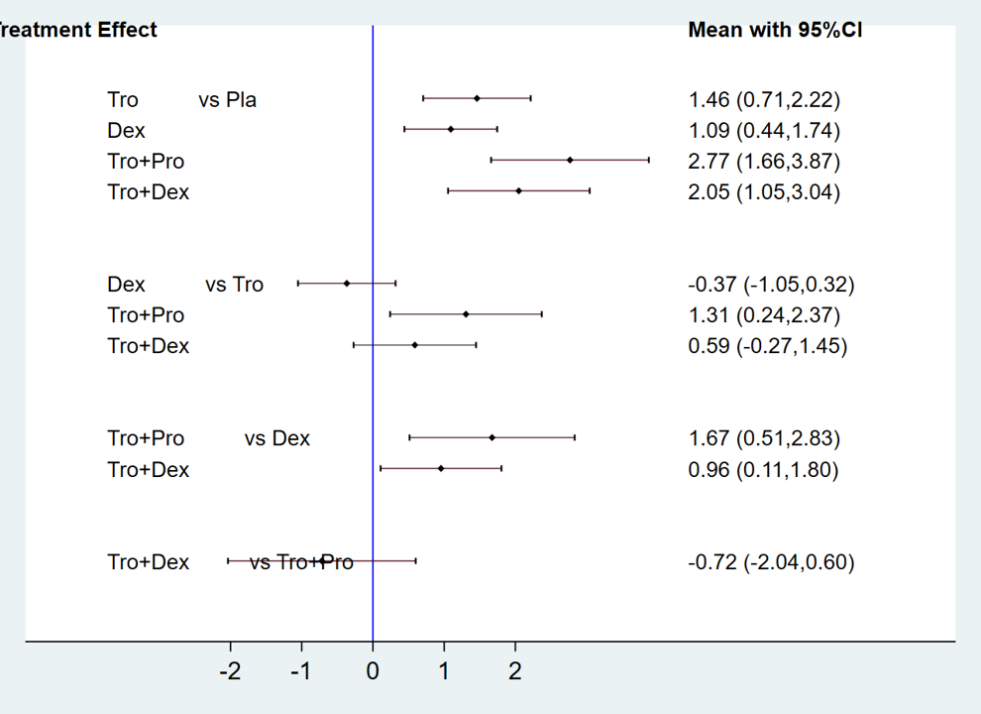


**S-Fig 6E. Predictive interval plots between each management modality for complete response.**

Diamond shape represents the mean summary effects. Black line represented the 95% CI, and red line represented the predictive interval (PrI). PrIs provide an interval that is expected to encompass the estimate of a future study.


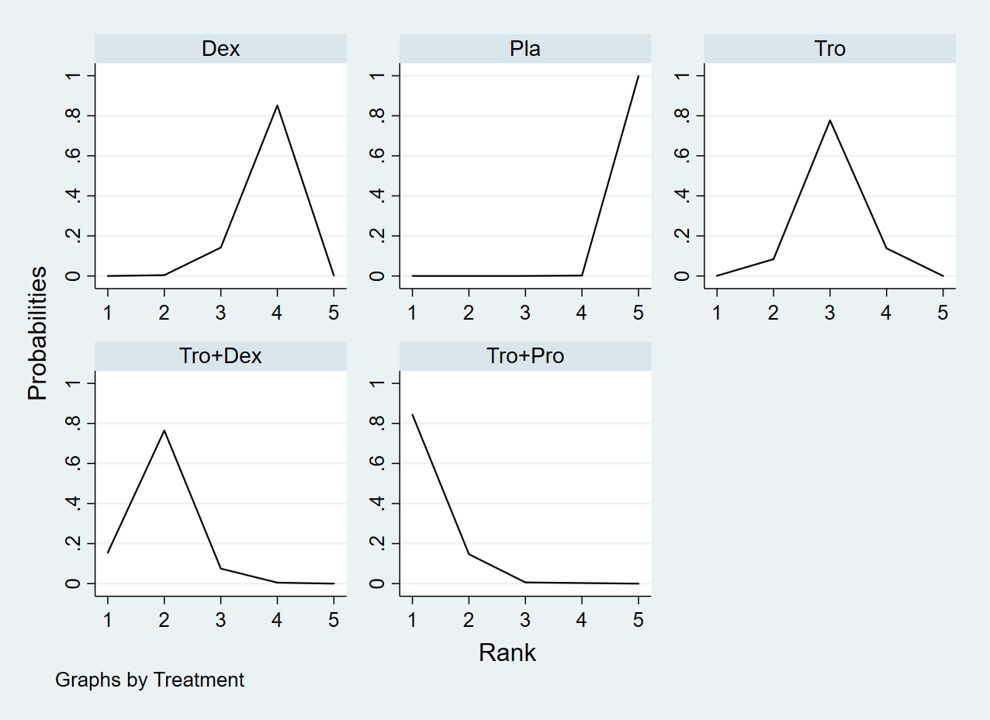


**S-Fig 7E. Rankogram for complete response.** Profiles indicate the probabilities for treatments to assume any of the possible ranks. It is the probability that a given treatment ranks first, second, third, and so on, among all of the treatments evaluated in the NMA.


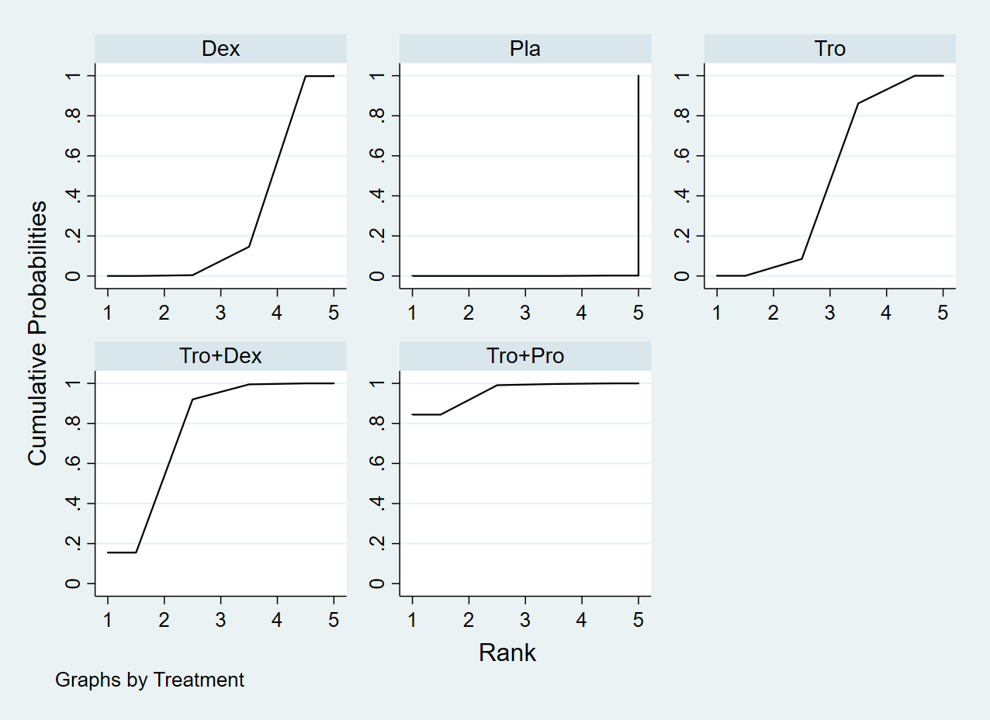


**S-Fig 8E. Cumulative ranking curve plot for complete response.** Profile indicates the sum of the probabilities from those ranked first, second, third, and so on. The surface under the cumulative ranking curve (SUCRA) value is regarded as an improved result for an individual’s intervention. When ranking treatments, the closer the SUCRA value is to 100%, the higher the treatment ranking is, relative to all other treatments.


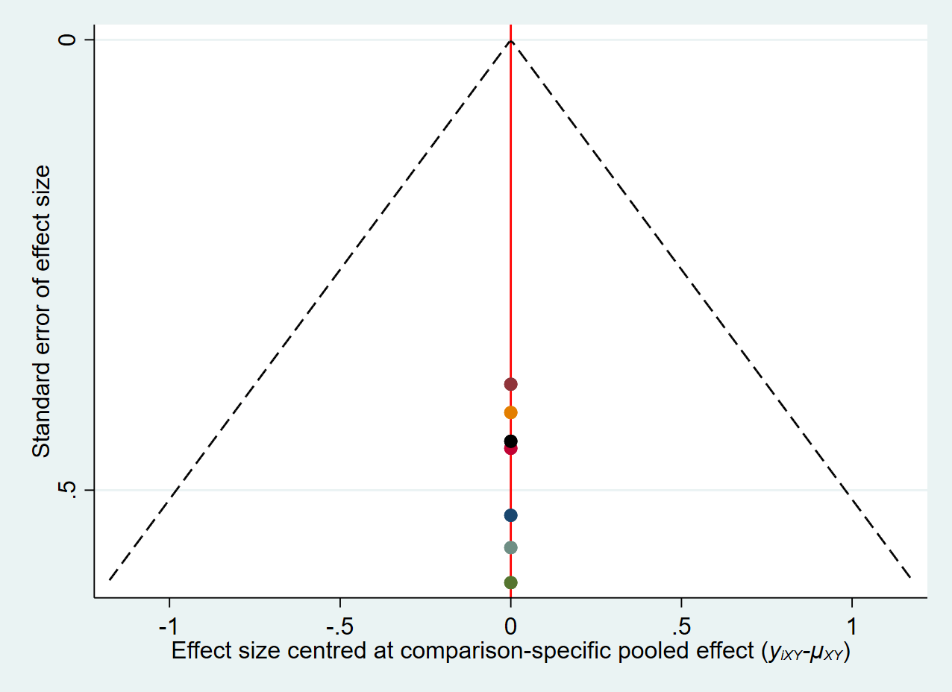


**S-Fig 9E. Comparison-adjusted funnel plot for complete response**
