# Supplementary material for: Pharmacologic interventions for postoperative nausea and vomiting after thyroidectomy: A systematic review and network meta-analysis
Source: PLoS One. 2021 Jan 11;16(1):e0243865. doi: 10.1371/journal.pone.0243865 (PMC7799806; doi:10.1371/journal.pone.0243865)
Supplement: S2 File — (DOCX) [file pone.0243865.s004.docx]

**Supplemental Files (2)**

**Table contents**

**1. Postoperative nausea and vomiting**

**1.1 Early phase**

**1.2 Middle phase**

**1.3 Late phase**

**2. Postoperative Nausea**

**2.1 Early phase**

**2.1.1 Mild**

**2.1.2 Moderate**

**2.2 Middle phase**

**2.2.1 Mild**

**2.2.2 Moderate**

**2.3 Late phase**

**2.3.1 Mild**

**2.3.2 Moderate**

**3. Postoperative vomiting**

**3.1 Early phase**

**3.2 Middle phase**

**3.3 Late phase**

**4. Use of rescue anti-emetics**

**4.1 Early phase**

**4.2 Middle phase**

**4.3 Late phase**

**5. Complete response**

**5.1 Early phase**

**5.2 Middle phase**

**5.3 Late phase**

For all outcomes of each specific datum, the summary of the results is presented from Figure 1 to Figure 8 (S-Fig a, b, c, d, e, f, g, h, i, j, k, l, m and n) correspond to early PONV, middle PONV, early PON-mild, early PON-moderate, middle PON-mild, middle PON-moderate, late PON-mild, late PON-moderate, early POV, middle POV, late POV, early use of rescue anti-emetics, middle use of rescue anti-emetics, and late use of rescue anti-emetics respectively).

We presented the network plot (Fig 1), inconsistency plot (Fig 2), confidence interval and/or predictive interval plot compared with placebo (Fig 3), rankogram (Fig 4), cumulative ranking curve (Fig 5), and expected mean ranking and SUCRA values of each pharmacologic agent for the outcomes (Fig 6) and comparison-adjusted funnel plot (Fig 7).

**Result**

1. **Postoperative nausea and vomiting**
   1. **Early phase**

S-Fig a1 depicts the network plot of the pharmacologic interventions comparing the PONV in the early phase. Eight pharmacologic interventions (Ond, Pro, Gra, Tro, Dex, Ram, Dro and Met) were compared in four studies. As four study were separated to two loops, we didn’t perform the NMA

- 1. **Middle phase**

S-Fig b1 depicts the network plot of the pharmacologic interventions comparing the PONV in the middle phase. Ten pharmacologic interventions (Ond, Pal, Pro, Gra, Tro, Dex, Pal+Dex, Ram, Dro and Met) were compared in five studies. As five study were separated to three loops, we didn’t perform the NMA

- 1. **Late phase**

Only one pharmacologic intervention (Dex) was compared in a study. So, we didn’t perform the NMA

1. **Postoperative Nausea**
   1. **Early phase**
      1. **Mild**

S-Fig c1 depicts the network plot of the pharmacological interventions comparing the incidence of mild Post-operative nausea (PON) in the early phase after thyroidectomy. Thirteen pharmacological interventions (Granisetron(Gra), Tropisetron+Dexamethasone(Tro+Dex), Ramosetron(Ram), Ondansetron(Ond), Propofol(Pro), Dexamenthasone(Dex), Palonosteron(Pal), Tropisetron(Tro), Palonosetron+Dexamethasone(Pal+Dex), Metoclopramide(Met), Droperidol(Dro), Intralipid(Int), Midazolam(Mid)) were compared in 15 studies for preventing incidence of mild PON in early phase.

The evaluation of the network inconsistency using the design-by-treatment interaction model suggested there was no evidence of inconsistency [F(8,11)=0.92 P=0.0167].

Of the nine closed loops in the network comparing the pharmacological interventions for mild PON in early phase, inconsistencies were suggested between the direct and indirect point estimates in the 2-7-14 loop (which included Ond-Tro-Met) and 2-6-7 loop (which included Ond-Gra-Tro). (S-Fig c2)

Gra, Tro+Dex, and Ram showed a lower incidence of PON than Control (Con) in terms of 95% confidence intervals (Cls). (S-Fig c3). The Rankogram showed that Gra had the lowest incidence of mild PON in the early phase (S-Fig c4).

The cumulative ranking plot was drawn, and the SUCRA probabilities of the different pharmacologic interventions for preventing the incidence of mild PON in early phase was calculated (S-Fig c5). The expected mean rankings and SUCRA values of each pharmacologic intervention were presented (S-Fig c6). According to the SUCRA value, the incidence of PON was the lowest when granisetron (11.1%) was used, followed by tropisetron combined with dexamethasone (18.8%), and followed by ramosetron (21.4%). The comparison-adjusted funnel plots showed that the funnel plots were symmetrical around the zero line, which suggested a less likely publication bias (S-Fig c7)

**2.1.2 Moderate**

A total of 8 studies comparing ten pharmacological interventions (Ram, Dex, Ond, Gra, Pal, Tro, Pal+Dex, Pro, Dro, Met) measured the incidence of moderate PON in the early phase after thyroidectomy. Of those, As one study comparing Pro, Dro and Met was separated from the loops, we performed the network metaanalysis(NMA) excluding that study. Thus, five studies comparing seven pharmacological intervention (Ram, Dex, Ond, Gra, Pal, Tro, Pal+Dex) were included in this NMA. (S-Fig d1)

There was no evidence of significance in the network inconsistency [F(2,5)=2.77 P=0.1533]. There was a closed loop in the network, which did not suggest inconsistency between the direct and indirect point estimates (S-Fig d2). Ram showed a lower incidence of moderate PON than Con and Tro in terms of the Cls (S-Fig d3).

The rankogram and cumulative ranking plot showed that Ram had the lowest incidence of moderate PON in early phase. (S-Fig d4 and S-Fig d5). The expected mean rankings and SUCRA plot showed that the incidence of moderate PON in the early phase was the lowest in Ram (5.5%), followed by Dex (34.8%), Ond (41.6%), Gra (42.8%), Palo (50.5%), Tro (71.7%), Con (72.1%), and Pal+Dex (80.9%) (S-Fig d6). The comparison-adjusted funnel plots suggested a less likely publication bias (S-Fig d7)

**2.2 Middle phase**

**2.2.1 Mild**

The network plot comparing the incidence of mild PON in the middle phase is depicted in S-Fig e1. Thirteen pharmacologic interventions (Pro, Int, Tro+Dex, Pal, Met, Dro, Mid, Pal+Dex, Tro, Ond, Gra, Ram, Dex) were compared in 15 studies.

There was a significant network inconsistency [F(8,11)=5.50 P=0.0057]. Of the nine closed loops in the network comparing the pharmacological interventions for mild PON in the middle phase, inconsistencies were suggested in the 2-6-7 loop (Ond-Gra-Tro) and 1-2-6 (Con-Ond-Gra), and 1-6-7 (Con-Gra-Tro). (S-Fig e2)

Pro, Tro+Dex, Pal, Met, Tro, Ond, Gra, and Ram showed a lower incidence of mild PON than Con in the middle phase (S-Fig e3).

The rankogram and cumulative ranking plot showed that Pro had the lowest incidence of mild PON in the middle phase (S-Fig e4 and S-Fig e5). The SUCRA plot displayed that the incidence of mild PON in the middle phase was the lowest in Pro (11.1%) (S-Fig e6). Publication bias was less likely in the comparison-adjusted funnel plot (S-Fig e7).

**2.2.2 Moderate**

A total of 7 studies comparing ten pharmacological interventions (Gra, Ram, Dex, Ond, Tro, Pal, Pal+Dex, Pro, Dro and Met) measured the incidence of moderate PON in the middle phase after thyroidectomy. Of those, as two studies comparing Pal vs. Pal+Dex and Pro vs. Dro vs. Met were separated from the loops, we performed the NMA excluding those studies. Thus, five studies comparing five pharmacological interventions (Gra, Ram, Dex, Ond, Tro) were included in this NMA. (S-Fig f1) The network plot of the incidence of mild PON in the middle phase is presented in S-Fig f2. There was no network inconsistency. [F(2,5)=4.08 P=0.0889]. A closed loop in the network that compared the incidence of moderate PON in the middle phase revealed no inconsistency between the direct and indirect point estimates (S-Fig f3).

The rankogram and cumulative ranking plot showed that Gra had the lowest incidence of mod PON in the middle phase (S-Fig f4 and S-Fig f5). The SUCRA plot and expected mean ranking showed that the incidence of mod PON in the middle phase was the lowest in Gra (22.2%), followed by Ram (30.2%), Dex (43.4%), Ond (50%) and Tro (75%) (S-Fig f6). The comparison-adjusted funnel plot implied a less likely publication bias (S-Fig f7).

**2.3 Late phase**

**2.3.1 Mild**

A total of 8 studies comparing eight pharmacological interventions (Gra, Tro, Dex, Ram, Dro, Mid, Int and Pro) measured the incidence of mild PON in the late phase after thyroidectomy. Of those, as two studies comparing Int vs. Pro and Dro vs. Mid were separated from the loops, we performed the NMA excluding those studies. Thus, six studies comparing seven pharmacological interventions (Gra, Tro, Dex and Ram) were included in this NMA. (S-Fig g1)

There was no network inconsistency. [F(2,4)=1.02 P=0.4389]. A closed loop in the network that compared the incidence of mild PON in the late phase revealed no inconsistency between the direct and indirect point estimates (S-Fig g2). Gra, Tro, Dex and Ram showed a lower incidence of mild PON than in terms of 95% Cls. (S-Fig g3).

The rankogram and cumulative ranking plot showed that Tro had the lowest incidence of mild PON in the late phase (S-Fig g4 and S-Fig g5). The SUCRA plot and expected mean ranking showed that the incidence of mild PON in the late phase was the lowest in Tro (6.8%), followed by Gra (35.1%) and Dex (45.1). (S-Figg6). The comparison-adjusted funnel plot implied a less likely publication bias (S-Fig g7).

**2.3.2 Moderate**

The network plot of the incidence of mod PON in the late phase is presented in S-Fig h1. Two pharmacologic interventions (Dex and Ram) were compared in 4 studies.

There was no network inconsistency. [F(2,2)=1.32 P=0.4317]. A closed loop in the network that compared the incidence of mild PON in the late phase revealed no inconsistency between the direct and indirect point estimates (S-Fig h2). Dex showed a lower incidence of mod PON than in terms of 95% Cls. (S-Fig h3).

The rankogram and cumulative ranking plot showed that Dex had the lowest incidence of mod PON in the late phase (S-Fig h4 and S-Fig h5). The SUCRA plot and expected mean ranking showed that the incidence of mild PON in the late phase was the lowest in Dex (15.3%) (S-Fig h6). The comparison-adjusted funnel plot implied a less likely publication bias (S-Fig h7).

1. **Postoperative Vomiting**
   1. **Early phase**

A total of 12 studies comparing thirteen pharmacological interventions (Gra, Tro, Dex, Tro+Dex, Ram, Mid, Dex+Gin, Ond, Pal, Met, Dro, Pro and Int) measured the incidence of POV in the early phase after thyroidectomy. Of those, as two studies comparing Pro vs. Int and Pro vs. Dro vs. Met were separated from the loops, we performed the NMA excluding those studies. Thus, ten studies comparing nine pharmacological interventions (Gra, Tro, Dex, Tro+Dex, Ram, Mid, Dex+Gin, Ond and Pal) were included in this NMA. (S-Fig i1)

The evaluation of the network inconsistency using the design-by-treatment interaction model suggested no significant inconsistency [F(5,9)=0.697 P=0.6438]. There were six closed loops in the network of the comparison of POV in the early phase. There was no significance in the local inconsistency between the direct and indirect point estimates (S-Fig i2). Dex+Gin, Tro+Dex, Pal, Gra, Ond, Dex, Ram and Tro showed a lower incidence of POV than Con in the early phase, which were significant only in terms of their 95% Cls and not in their 95% Prls (S-Fig i3).

The rankogram showed that Dex+Gin had the lowest incidence of POV in the early phase (S-Fig i4). The cumulative ranking plot was drawn, and the SUCRA probabilities of the different pharmacological intervention for reducing POV in the early phase were calculated (S-Fig i5). The expected mean rankings and SUCRA values of each pharmacological intervention were presented (S-Fig i6). According to SUCRA value, the POV was the lowest in Dex+Gin (8.5%), Tro+Dex (20.1%), Pal (33.4%), Gra (42.2%), Ond (44.9%), and Dex (45.9%). The comparison-adjusted funnel plots showed that the funnel plots were symmetrical around the zero line, which suggested a less likely publication bias (S-Fig i7).

- 1. **Middle phase**

A total of 13 studies comparing 14 pharmacological interventions (Gra, Tro, Dex, Tro+Dex, Pal+Dex, Ram, Dro, Mid, Dex+Gin, Met, Ond, Pal, Pro and Int) measured the incidence of POV in the middle phase after thyroidectomy. Of those, as two studies comparing Pro vs. Int and Pro vs. Dro vs. Met were separated from the loops, we performed the NMA excluding those studies Thus, 11 studies comparing 10 pharmacological interventions (Gra, Tro, Dex, Tro+Dex, Pal+Dex, Ram, Mid, Dex+Gin, Ond and Pal) were included in this NMA. (S-Fig j1)

The evaluation of the network inconsistency using the design-by-treatment interaction model suggested no significant inconsistency [F(5,9)=1.85 P=0.1995]. There were six closed loops in the network of the comparison of POV in the early phase. There was no significance in the local inconsistency between the direct and indirect point estimates (S-Fig j2).

Pal, Tro+Dex, Dex+Gin, Ond, Pal+Dex, Ram, Gra, Dex, and Tro showed a lower incidence of POV than Con in the middle phase, which were significant only in terms of their 95% Cls and not in their 95% Prls (S-Fig j3).

The rankogram showed that Pal had the lowest incidence of POV in the middle phase (S-Fig j4). The cumulative ranking plot was drawn, and the SUCRA probabilities of the different pharmacological intervention for reducing POV in the middle phase were calculated (S-Fig j5). The expected mean rankings and SUCRA values of each pharmacological intervention were presented (S-Fig j6). According to SUCRA value, the POV was the lowest in Pal(14.2%), Tro+Dex (16.4%), Dex+Gin (27.8%), Ond (40.3%), Ram (44.5%) and Pal+Dex (46.6%). The comparison-adjusted funnel plots showed that the funnel plots were symmetrical around the zero line, which suggested a less likely publication bias (S-Fig j7).

- 1. **Late phase**

A total of 6 studies comparing 4 pharmacological interventions (Dex, Ram, Int and Pro) measured the incidence of POV in the late phase after thyroidectomy. Of those, one study comparing Pro vs. Int was separated from the loops, we performed the NMA excluding those studies. Thus, 5 studies comparing 2 pharmacological interventions (Dex and Ram) were included in this NMA. (S-Fig k1)

The evaluation of the network inconsistency using the design-by-treatment interaction model suggested no significant inconsistency [F(2,3)=0.11 P=0.8983]. There was one closed loop in the network of the comparison of POV in the late phase. There was no significance in the local inconsistency between the direct and indirect point estimates (S-Figk2).

Con showed a lower incidence of POV than Ram and Dex in the late phase, which was only in terms of their 95% Cls and not in their 95% Prls. Ram showed a lower incidence of POV in the late phase Dex only in terms of 95% Cls (S-Fig k3).

The rankogram showed that Con had the lowest incidence of POV in the late phase (S-Fig k4). The cumulative ranking plot was drawn, and the SUCRA probabilities of the different pharmacological intervention for reducing POV in the late phase were calculated (S-Fig k5). The expected mean rankings and SUCRA values of each pharmacological intervention were presented (S-Fig k6). According to SUCRA value, the POV was the lowest in Con (34.9%). The comparison-adjusted funnel plots showed that the funnel plots were symmetrical around the zero line, which suggested a less likely publication bias (S-Fig k7).

1. **Use of Rescue Antiemetics**
   1. **Early phase**

A total of 8 studies comparing 11 pharmacological interventions (Ond, Pal, Pro, Gra, Tro Dex, Ram, Dro, Mid, Dex+Gin and Met) measured the use of rescue antiemetics in the early phase after thyroidectomy. As three studies comparing Pal vs. Ond and Pro vs. Dro vs. Met and Ond vs. Tro vs. Met were separated from the loops, we performed the NMA excluding those studies. Thus, 3 studies comparing 5 pharmacological interventions (Gra, Dex, Ram, Mid and Dex+Gin) were included in this NMA. (S-Fig l1)

The evaluation of the network inconsistency using the design-by-treatment interaction model suggested no significant inconsistency [F(3,3)=1.36 P=0.4043]. There were two closed loops in the network of the comparison of use of rescue antiemetics in the early phase. There was no significance in the local inconsistency between the direct and indirect point estimates (S-Fig l2).

Dex+Gin, Gra, Ram, Mid and Dex showed a lower use of rescue antiemetics than Con in the early phase only in terms of 95% their Cls and not in their 95% Prls (S-Fig l3).

The rankogram and cumulative ranking plot showed Dex+Gin to be the most effective pharmacological intervention in terms of reducing use of antiemetics (S-Fig l4 and S-Fig l5). The expected mean rankings and the SUCRA plots showed that the use of antiemetics was the lowest in Dex+Gin (10.5%), followed by Ram (29.8%), and Gra (32.1%) (S-Fig l6). The comparison-adjusted funnel plots suggested a less likely publication bias (S-Fig l7)

- 1. **Middle phase**

A total of 8 studies comparing 11 pharmacological interventions (Ond, Pal, Pro, Gra, Tro, Dex, Ram, Dro, Mid, Dex+Gin and Met) measured the use of rescue antiemetics in the early phase after thyroidectomy. As three studies comparing Pal vs. Ond and Pro vs. Dro vs. Met and Ond vs. Tro vs. Met were separated from the loops, we performed the NMA excluding those studies. Thus, 3 studies comparing 5 pharmacological interventions (Gra, Dex, Ram, Mid and Dex+Gin) were included in this NMA (S-Fig m1)

The evaluation of the network inconsistency using the design-by-treatment interaction model suggested no significant inconsistency [F(3,3)=1.87 P=0.3095]. There were two closed loops in the network of the comparison of use of rescue antiemetics in the middle phase. There was no significance in the local inconsistency between the direct and indirect point estimates (S-Fig m2).

Gra, Ram, Dex+Gin, Dex showed a lower use of rescue antiemetics than Con in the middle phase only in terms of 95% their Cls and not in their 95% Prls (S-Fig m3).

The rankogram and cumulative ranking plot showed Gra to be the most effective pharmacological intervention in terms of reducing use of antiemetics (S-Fig m4 and S-Fig m5). The expected mean rankings and the SUCRA plots showed that the use of antiemetics was the lowest in Gra (20.9%), followed by Ram (33%), and Dex+Gin (39.1%) (S-Fig m6). The comparison-adjusted funnel plots suggested a less likely publication bias (S-Fig m7)

- 1. **Late phase**

Two pharmacologic interventions (Dex and Ram) were compared in 3 studies (S-Fig n1).

The evaluation of the network inconsistency using the design-by-treatment interaction model suggested inconsistency because of insufficient observation (S-Fig n2).

Dex and Ram showed a lower use of rescue antiemetics than Con in the late phase only in terms of 95% their Cls and not in their 95% Prls (S-Fig n3).

The rankogram and cumulative ranking plot showed Dex to be the most effective pharmacological intervention in terms of reducing use of antiemetics (S-Fig n4 and S-Fig n5). The expected mean rankings and the SUCRA plots showed that the use of antiemetics was the lowest in Dex (24.9%) (S-Fig n6). The comparison-adjusted funnel plots suggested a less likely publication bias (S-Fig n7)

1. **Complete Response**
   1. **Early phase**

Three pharmacologic interventions (Pal, Pal+Dex and Ram) were compared in two studies. As two study were separated to two loops, we didn’t perform the NMA

- 1. **Middle phase**

Three pharmacologic interventions (Pal, Pal+Dex and Ram) were compared in two studies. As two study were separated to two loops, we didn’t perform the NMA

- 1. **Late phase**

Only one pharmacologic intervention (Ram) was compared in a study. So we didn’t perform the NMA

**Fig 1. Network plot of included studies comparing different pharmacological strategies.** The nodes show a comparison of pharmacological regimens to prevent PON, POV, PONV, use of rescue anti-emetics and complete response, and the edges show the available direct comparisons among the pharmacological regimens. The nodes and edges are weighed on the basis of the number of included patients and inverse of standard error of effect.

**Fig 2. Inconsistency plot between the direct and indirect effect estimates for the same comparison**. Inconsistency factor (IF) as the absolute difference with 95% confidence interval (CI) between the direct and indirect estimates for each paired comparison is presented. IF values close to 0 indicate that the two sources are in agreement.

**Fig 3. Confidence interval and/or predictive interval plots between each management modality and placebo group. group.** Diamond shape represents the mean summary effects. Black line represented the 95% CI, and red line represented the predictive interval (PrI). PrIs provide an interval that is expected to encompass the estimate of a future study.

**Fig 4. Rankogram.** Profiles indicate the probabilities for treatments to assume any of the possible ranks. It is the probability that a given treatment ranks first, second, third, and so on, among all of the treatments evaluated in the NMA.

**Fig 5. Cumulative ranking curve plot.** Profile indicates the sum of the probabilities from those ranked first, second, third, and so on. A higher cumulative ranking curve (SUCRA) value is regarded as an improved result for an individual’s intervention. When ranking treatments, the closer the SUCRA value is to 100%, the higher the treatment ranking is, relative to all other treatments.

**Fig6. Expected mean ranking and SUCRA values.** X-axis corresponds to expected mean ranking based on SUCRA (surface of under cumulative ranking curve) value, and Y-axis corresponds to SUCRA value.

**Fig 7. comparison-adjusted funnel plot**
