# Supplementary material for: Pharmacologic interventions for postoperative nausea and vomiting after thyroidectomy: A systematic review and network meta-analysis
Source: PLoS One. 2021 Jan 11;16(1):e0243865. doi: 10.1371/journal.pone.0243865 (PMC7799806; doi:10.1371/journal.pone.0243865)
Supplement: S1 Table — (DOCX) [file pone.0243865.s005.docx]

**Supplementary Table 1: Transitivity assessment**

| **Study  (1^st^ author, year)** | **Patient eligibility criteria** | **Gender**  **(F/M)** | **Age** | **Types of pharmacologic interventions** | **Study design** | **ROB** | | |
| --- | --- | --- | --- | --- | --- | --- | --- | --- |
|  |  |  |  |  |  | **Overall risk of bias judgement** | **Bias arising from the randomization process** | **Bias in measurement of the outcome** |
| ***PONV*** |  |  |  |  |  |  |  |  |
| Moon YE, 2012 | ASA I-II  Undergo total thyroidectomy | Female only | 20-60yr | Ond 8mg bolus and 16mg in IV PCA | RCT | Low risk | Low risk | Low risk |
|  |  |  |  | Pal 0.075mg IV |  |  |  |  |
| Ewalenk P, 1996 | ASA I-II  Undergo thyroidectomy | Both sex (57/7) | 22-71yr | Pro 0.1mg/kg/hr IV | RCT | Some concerns | Some concerns | Low risk |
|  |  |  |  | 10% Int 0.1mg/kg/hr IV |  |  |  |  |
| Song YK, 2013 | ASA I-II  Undergo thyroid surgery | Female only | 20-65yr | Pla | RCT | Some concerns | Low risk | Some concerns |
|  |  |  |  | Dex 10mg IV |  |  |  |  |
|  |  |  |  | Ram 0.3mg IV |  |  |  |  |
| Lee SY, 2002 | ASA I-II  Undergo thyroidectomy | Both sex (104/9) | 18-65yr | Pla | RCT | Some concerns | Some concerns | Low risk |
|  |  |  |  | Gra 20μg/kg IV |  |  |  |  |
|  |  |  |  | Ram 4μg/kg IV |  |  |  |  |
| Wang JJ, 1999 | Undergo partial or total thyroidectomy | Female only | Mean  41 | Dex 10mg IV | RCT | Some concerns | Some concerns | Low risk |
|  |  |  | 38 | Dro 1.25mg IV |  |  |  |  |
|  |  |  | 42 | Pla |  |  |  |  |
| Worni M, 2008 | Undergo thyroid surgery | Both sex (53/19) | 18-80yr | P | RCT | Some concerns | Some concerns | Low risk |
|  |  |  |  | Dex 8mg IV |  |  |  |  |
| Fujii Y, 2001 | ASA I  Undergo thyroidectomy | Both sex (75/15) | 33-58yr | Pro 0.5mg.kg IV | RCT | Some concerns | Some concerns | Low risk |
|  |  |  |  | Dro 20μg/kg IV |  |  |  |  |
|  |  |  |  | Met 0.2mg/kg IV |  |  |  |  |
| Jokela R, 2002 | ASA I-III  Undergo thyroid or parathyroid surgery | Female only | Mean  51 | Ond 16mg IV | RCT | Some concerns | Low risk | Some concerns |
|  |  |  | 49 | Tro 5mg IV |  |  |  |  |
|  |  |  | 47 | Met 10mg IV |  |  |  |  |
| ***PON*** |  |  |  |  |  |  |  |  |
| Moon YE, 2012 | ASA I-II  Undergo total thyroidectomy | Female only | 20-60yr | Ond 8mg bolus and 16mg in IV PCA | RCT | Low risk | Low risk | Low risk |
|  |  |  |  | Pal 0.075mg IV |  |  |  |  |
| Jeon Y, 2010 | ASA I-II  Undergo thyroidectomy | Female | Mean 46.5yr | Ram 0.3mg IV | RCT | Low risk | Low risk | Low risk |
|  |  |  |  | Dex 8mg IV |  |  |  |  |
|  |  |  |  | Ram 0.3mg + Dex 8mg IV |  |  |  |  |
| Eberhar LH, 1999 | ASA I-III  Undergo thyroidectomy or laparoscopic cholecystectomy | Both sex (102/48) | 20-64yr | Dro 5-7.5mg IV | RCT | Some concerns | Some concerns | Low risk |
|  |  |  |  | Mid 5-7.5mg IV *5mg: body weight<70kg,  7.5mg: body weight≥70kg |  |  |  |  |
| Song YK, 2013 | ASA I-II  Undergo thyroid surgery | Female only | 20-65yr | Pla | RCT | Some concerns | Low risk | Some concerns |
|  |  |  |  | Dex 10mg IV |  |  |  |  |
|  |  |  |  | Ram 0.3mg IV |  |  |  |  |
| Akin A, 2006 | Undergo thyroid surgery | Both sex (87/18) | 19-68yr | Tro 5mg IV | RCT | High risk | Some concerns | Some concerns |
|  |  |  |  | Tro 5mg + Pro 0.5mg/kg IV |  |  |  |  |
|  |  |  |  | Pla |  |  |  |  |
| Fujii Y, 2007 | ASA I-II  Undergo thyroidectomy | Both sex (53/22) | 32-58yr | Pla | RCT | Some concerns | Some concerns | Low risk |
|  |  |  |  | Dex 4mg IV |  |  |  |  |
|  |  |  |  | Dex 8mg IV |  |  |  |  |
| Tavlan A, 2006 | ASA I-II  Undergo thyroidectomy | Both sex (106/14) | Mean 39.5 | Dex | RCT | Low risk | Low risk | Low risk |
|  |  |  | 38.5 | Dex + Gin 0.5g oral |  |  |  |  |
| Lee SY, 2002 | ASA I-II  Undergo thyroidectomy | Both sex (104/9) | 18-65yr | Pla | RCT | Some concerns | Some concerns | Low risk |
|  |  |  |  | Gra 20μg/kg IV |  |  |  |  |
|  |  |  |  | Ram 4μg/kg IV |  |  |  |  |
| Wang JJ, 1999 | Undergo partial or total thyroidectomy | Female only | Mean 41 | Dex 10mg IV | RCT | Some concerns | Some concerns | Low risk |
|  |  |  | 38 | Dro 1.25mg IV |  |  |  |  |
|  |  |  | 42 | Pla |  |  |  |  |
| Wang JJ, 2000 | Undergo partial or total thyroidectomy | Female only | Mean  42 | Dex10mg | RCT | Some concerns | Some concerns | Low risk |
|  |  |  | 41 | Dex 5mg |  |  |  |  |
|  |  |  | 42 | Dex 2.5mg |  |  |  |  |
|  |  |  | 38 | Dex 1.25mg |  |  |  |  |
|  |  |  | 41 | Pla |  |  |  |  |
| Fujii Y, 2001 | ASA I  Undergo thyroidectomy | Both sex (75/15) | 33-58yr | Pro 0.5mg.kg IV | RCT | Some concerns | Some concerns | Low risk |
|  |  |  |  | Dro 20μg/kg IV |  |  |  |  |
|  |  |  |  | Met 0.2mg/kg IV |  |  |  |  |
| Jokela R, 2002 | ASA I-III  Undergo thyroid or parathyroid surgery | Female only | Mean  51 | Ond 16mg IV | RCT | Some concerns | Low risk | Some concerns |
|  |  |  | 49 | Tro 5mg IV |  |  |  |  |
|  |  |  | 47 | Met 10mg IV |  |  |  |  |
| Lee MJ, 2015 | ASA I-II  Undergo thyroid surgery | Both sex (93/15) | 20-65yr | Pla | RCT | Low risk | Low risk | Low risk |
|  |  |  |  | Ram 0.3mg |  |  |  |  |
|  |  |  |  | Ram 0.3mg + Dex 5mg |  |  |  |  |
| ***POV*** |  |  |  |  |  |  |  |  |
| Jeon Y, 2010 | ASA I-II  Undergo thyroidectomy | Female only | Mean 46.5yr | Ram 0.3mg IV | RCT | Low risk | Low risk | Low risk |
|  |  |  |  | Dex 8mg IV |  |  |  |  |
|  |  |  |  | Ram 0.3mg + Dex 8mg IV |  |  |  |  |
| Song YK, 2013 | ASA I-II  Undergo thyroid surgery | Female only | 20-65yr | Pla | RCT | Some concerns | Low risk | Some concerns |
|  |  |  |  | Dex 10mg IV |  |  |  |  |
|  |  |  |  | Ram 0.3mg IV |  |  |  |  |
| Akin A, 2006 | Undergo thyroid surgery | Both sex (87/18) | 19-68yr | Tro 5mg IV | RCT | High risk | Some concerns | Some concerns |
|  |  |  |  | Tro 5mg + Pro 0.5mg/kg IV |  |  |  |  |
|  |  |  |  | Pla |  |  |  |  |
| Fujii Y, 2007 | ASA I-II  Undergo thyroidectomy | Both sex (53/22) | 32-58yr | Pla | RCT | Some concerns | Some concerns | Low risk |
|  |  |  |  | Dex 4mg IV |  |  |  |  |
|  |  |  |  | Dex 8mg IV |  |  |  |  |
| Papadima A, 2013 | ASA I-II  Undergo thyroidectomy | Both sex (100/27) | 18-75yr | Gra 3mg IV | RCT | Some concerns | Some concerns | Low risk |
|  |  |  |  | Tro 5mg IV |  |  |  |  |
|  |  |  |  | Pla |  |  |  |  |
| Tavlan A, 2006 | ASA I-II  Undergo thyroidectomy | Both sex (106/14) | Mean 39.5 | Dex | RCT | Low risk | Low risk | Low risk |
|  |  |  | 38.5 | Dex + Gin 0.5g oral |  |  |  |  |
| Lee SY, 2002 | ASA I-II  Undergo thyroidectomy | Both sex (104/9) | 18-65yr | Pla | RCT | Some concerns | Some concerns | Low risk |
|  |  |  |  | Gra 20μg/kg IV |  |  |  |  |
|  |  |  |  | Ram 4μg/kg IV |  |  |  |  |
| Wang JJ, 1999 | Undergo partial or total thyroidectomy | Female only | Mean  41 | Dex 10mg IV | RCT | Some concerns | Some concerns | Low risk |
|  |  |  | 38 | Dro 1.25mg IV |  |  |  |  |
|  |  |  | 42 | Pla |  |  |  |  |
| Lee MJ, 2015 | ASA I-II  Undergo thyroid surgery | Both sex (93/15) | 20-65yr | Pla | RCT | Low risk | Low risk | Low risk |
|  |  |  |  | Ram 0.3mg |  |  |  |  |
|  |  |  |  | Ram 0.3mg + Dex 5mg |  |  |  |  |
| ***Use of antiemetics*** | | | | | | | | |
| Ewalenk P, 1996 | ASA I-II  Undergo thyroidectomy | Both sex (57/7) | 22-71yr | Pro 0.1mg/kg/hr IV | RCT | Some concerns | Some concerns | Low risk |
|  |  |  |  | 10% Int 0.1mg/kg/hr IV |  |  |  |  |
| Zhou H, 2012 | Undergo thyroid surgery | Both sex (114/36) | >18yr | Dex 8mg IV | RCT | Some concerns | Some concerns | Low risk |
|  |  |  |  | Tro 5mg IV |  |  |  |  |
|  |  |  |  | Dex 8mg + Tro 5mg IV |  |  |  |  |
| Jeon Y, 2010 | ASA I-II  Undergo thyroidectomy | Female only | Mean 46.5yr | Ram 0.3mg IV | RCT | Low risk | Low risk | Low risk |
|  |  |  |  | Dex 8mg IV |  |  |  |  |
|  |  |  |  | Ram 0.3mg + Dex 8mg IV |  |  |  |  |
| Barros A, 2013 | ASA I-III  Undergo thyroidectomy | Female only | 18-75yr | Dex 4mg IV | RCT | Some concerns | Some concerns | Low risk |
|  |  |  |  | Pla |  |  |  |  |
| Schietrom M, 2013 | ASA I-II  Undergo total thyroidectomy or lobectomy | Both sex (198/130) | 25-67yr | Dex 8mg IV | RCT | Low risk | Low risk | Low risk |
|  |  |  |  | Pla |  |  |  |  |
| Eberhar LH, 1999 | ASA I-III  Undergo thyroidectomy or laparoscopic cholecystectomy | Both sex (102/48) | 20-64yr | Dro 5-7.5mg IV | RCT | Some concerns | Some concerns | Low risk |
|  |  |  |  | Mid 5-7.5mg IV *5mg: body weight<70kg,  7.5mg: body weight≥70kg |  |  |  |  |
| Song YK, 2013 | ASA I-II  Undergo thyroid surgery | Female only | 20-65yr | Pla | RCT | Some concerns | Low risk | Some concerns |
|  |  |  |  | Dex 10mg IV |  |  |  |  |
|  |  |  |  | Ram 0.3mg IV |  |  |  |  |
| Akin A, 2006 | Undergo thyroid surgery | Both sex (87/18) | 19-68yr | Tro 5mg IV | RCT | High risk | Some concerns | Some concerns |
|  |  |  |  | Tro 5mg + Pro 0.5mg/kg IV |  |  |  |  |
|  |  |  |  | Pla |  |  |  |  |
| Papadima A, 2013 | ASA I-II  Undergo thyroidectomy | Both sex (100/27) | 18-75yr | Gra 3mg IV | RCT | Some concerns | Some concerns | Low risk |
|  |  |  |  | Tro 5mg IV |  |  |  |  |
|  |  |  |  | Pla |  |  |  |  |
| Tavlan A, 2006 | ASA I-II  Undergo thyroidectomy | Both sex (106/14) | Mean 39.5 | Dex | RCT | Low risk | Low risk | Low risk |
|  |  |  | 38.5 | Dex + Gin 0.5g oral |  |  |  |  |
| Lee SY, 2002 | ASA I-II  Undergo thyroidectomy | Both sex (104/9) | 18-65yr | Pla | RCT | Some concerns | Some concerns | Low risk |
|  |  |  |  | Gra 20μg/kg IV |  |  |  |  |
|  |  |  |  | Ram 4μg/kg IV |  |  |  |  |
| Zhang HW, 2016 | ASA I-II  Undergo thyroid surgery | Both sex (180/53) | 20-65yr | Dex 0.1mg/kg IV | RCT | Some concerns | Some concerns | Low risk |
|  |  |  |  | Pla |  |  |  |  |
| Wang JJ, 2000 | Undergo partial or total thyroidectomy | Female only | Mean  42 | Dex10mg | RCT | Some concerns | Some concerns | Low risk |
|  |  |  | 41 | Dex 5mg |  |  |  |  |
|  |  |  | 42 | Dex 2.5mg |  |  |  |  |
|  |  |  | 38 | Dex 1.25mg |  |  |  |  |
|  |  |  | 41 | Pla |  |  |  |  |
| Fujii Y, 2001 | ASA I  Undergo thyroidectomy | Both sex (75/15) | 33-58yr | Pro 0.5mg.kg IV | RCT | Some concerns | Some concerns | Low risk |
|  |  |  |  | Dro 20μg/kg IV |  |  |  |  |
|  |  |  |  | Met 0.2mg/kg IV |  |  |  |  |
| Jokela R, 2002 | ASA I-III  Undergo thyroid or parathyroid surgery | Female only | Mean  51 | Ond 16mg IV | RCT | Some concerns | Low risk | Some concerns |
|  |  |  | 49 | Tro 5mg IV |  |  |  |  |
|  |  |  | 47 | Met 10mg IV |  |  |  |  |
| Lee MJ, 2015 | ASA I-II  Undergo thyroid surgery | Both sex (93/15) | 20-65yr | Pla | RCT | Low risk | Low risk | Low risk |
|  |  |  |  | Ram 0.3mg |  |  |  |  |
|  |  |  |  | Ram 0.3mg + Dex 5mg |  |  |  |  |
| ***Complete response*** | | | | | | | | |
| Zhou H, 2012 | Undergo thyroid surgery | Both sex (114/36) | >18yr | Dex 8mg IV | RCT | Some concerns | Some concerns | Low risk |
|  |  |  |  | Tro 5mg IV |  |  |  |  |
|  |  |  |  | Dex 8mg + Tro 5mg IV |  |  |  |  |
| Park JW, 2012 | ASA I-II  undergo laparoscopic gynecologic surgery, mastoidectomy with tympanoplasty or thyroidectomy. | N/R | 18-60yr | Pal 0.075mg IV | RCT | High risk | Some concerns | Some concerns |
|  |  |  |  | Pal 0.075mg + Dex 4mg IV |  |  |  |  |
|  |  |  |  | Dex 10mg IV |  |  |  |  |
|  |  |  |  | Ram 0.3mg IV |  |  |  |  |
| Akin A, 2006 | Undergo thyroid surgery | Both sex (87/18) | 19-68yr | Tro 5mg IV | RCT | High risk | Some concerns | Some concerns |
|  |  |  |  | Tro 5mg + Pro 0.5mg/kg IV |  |  |  |  |
|  |  |  |  | Pla |  |  |  |  |
| Wang JJ, 2000 | Undergo partial or total thyroidectomy | Female only | Mean  42 | Dex10mg | RCT | Some concerns | Some concerns | Low risk |
|  |  |  | 41 | Dex 5mg |  |  |  |  |
|  |  |  | 42 | Dex 2.5mg |  |  |  |  |
|  |  |  | 38 | Dex 1.25mg |  |  |  |  |
|  |  |  | 41 | Pla |  |  |  |  |

PONV: post-operative nausea and vomiting; IV: intravenous; Int: Intralipid; Ond: ondansetron; Pal: palonosetron; PCA: patient-controlled analgesia; IM: intramuscular; Pla: placebo; Gra: granisetron; Tro: tropisetron; Dex: dexamethasone; Pro: proprofol; Dia: diazepam; Ram: ramosetron; Dro: droperidol; Mid: midazolam; VAS: visual analogue pain score; TCI: target-controlled infusion; PAS: postanesthetic shivering; TCI: target-controlled infusion; SC: subcutaneous; Met: metoclopramide; Clon: clonidine; Gin: oral ginger; RCT: Randomized control trial; N/R: not reported
