# Supplementary material for: Pharmacologic interventions for postoperative nausea and vomiting after thyroidectomy: A systematic review and network meta-analysis
Source: PLoS One. 2021 Jan 11;16(1):e0243865. doi: 10.1371/journal.pone.0243865 (PMC7799806; doi:10.1371/journal.pone.0243865)
Supplement: S2 Table — (DOCX) [file pone.0243865.s006.docx]

**Supplementary Table 2. Safety**

| Study  (1^st^ author, year) | Interventions | Sample size | Safety | | | | | | | | | | | | | | | | |  |
| --- | --- | --- | --- | --- | --- | --- | --- | --- | --- | --- | --- | --- | --- | --- | --- | --- | --- | --- | --- | --- |
|  |  |  | Headache | Dizziness | Drowsiness | Constipation | HTN | Wound infection | Dyspepsia | Delayed Wound healing | Coughing | Tachycardia | Bladder distension | Allergic reaction | Hypocalcemia | Bradycardia | Pruritis | Visual disturbance | Myocardial infarction | |
| Moon YE, 2012 | Ond 8mg bolus and 16mg in IV PCA | 50 | 19 | 11 | 6 | N/R | N/R | N/R | N/R | N/R | N/R | N/R | N/R | N/R | N/R | N/R | N/R | N/R | N/R | |
|  | Pal 0.075mg | 50 | 18 | 7 | 5 | N/R | N/R | N/R | N/R | N/R | N/R | N/R | N/R | N/R | N/R | N/R | N/R | N/R | N/R | |
| Ewalenk P, 1996 | Pro 0.1mg/kg/hr IV | 32 | N/R | N/R | N/R | N/R | N/R | N/R | N/R | N/R | N/R | N/R | N/R | N/R | N/R | N/R | N/R | N/R | N/R | |
|  | 10% Int 0.1mg/kg/hr IV | 32 | N/R | N/R | N/R | N/R | N/R | N/R | N/R | N/R | N/R | N/R | N/R | N/R | N/R | N/R | N/R | N/R | N/R | |
| Metaxari M, 2011 | Pla 5mg | 50 | N/R | N/R | N/R | N/R | N/R | N/R | N/R | N/R | N/R | N/R | N/R | N/R | N/R | N/R | N/R | N/R | N/R | |
|  | Gra 3mg | 50 | N/R | N/R | N/R | N/R | N/R | N/R | N/R | N/R | N/R | N/R | N/R | N/R | N/R | N/R | N/R | N/R | N/R | |
|  | Ond 4mg | 51 | N/R | N/R | N/R | N/R | N/R | N/R | N/R | N/R | N/R | N/R | N/R | N/R | N/R | N/R | N/R | N/R | N/R | |
|  | Tro 5mg | 52 | N/R | N/R | N/R | N/R | N/R | N/R | N/R | N/R | N/R | N/R | N/R | N/R | N/R | N/R | N/R | N/R | N/R | |
| Zhou H, 2012 | Dex 8mg | 50 | 4 | 3 | N/R | 0 | N/R | N/R | N/R | N/R | N/R | N/R | N/R | N/R | N/R | N/R | N/R | N/R | N/R | |
|  | Tro 5mg | 50 | 4 | 4 | N/R | 1 | N/R | N/R | N/R | N/R | N/R | N/R | N/R | N/R | N/R | N/R | N/R | N/R | N/R | |
|  | Dex 8mg +Tro 5mg | 50 | 3 | 4 | N/R | 1 | N/R | N/R | N/R | N/R | N/R | N/R | N/R | N/R | N/R | N/R | N/R | N/R | N/R | |
| Park JW, 2012 | Pal 0.075mg | 41 | N/R | N/R | N/R | N/R | N/R | N/R | N/R | N/R | N/R | N/R | N/R | N/R | N/R | N/R | N/R | N/R | N/R | |
|  | Pal 0.075mg + Dex 4mg | 43 | N/R | N/R | N/R | N/R | N/R | N/R | N/R | N/R | N/R | N/R | N/R | N/R | N/R | N/R | N/R | N/R | N/R | |
| Jeon Y, 2010 | Ram 0.3mg | 60 | 12 | 13 | N/R | 1 | N/R | N/R | N/R | N/R | N/R | N/R | N/R | N/R | N/R | N/R | N/R | N/R | N/R | |
|  | Dex 8mg | 60 | 9 | 11 | N/R | 2 | N/R | N/R | N/R | N/R | N/R | N/R | N/R | N/R | N/R | N/R | N/R | N/R | N/R | |
|  | Ram 0.3mg + Dex 8mg | 60 | 11 | 9 | N/R | 2 | N/R | N/R | N/R | N/R | N/R | N/R | N/R | N/R | N/R | N/R | N/R | N/R | N/R | |
| Doksrod S, 2012 | Dex 0.3mg/kg | 40 | N/R | N/R | N/R | N/R | N/R | 2 | 1 | 1 | N/R | N/R | N/R | N/R | N/R | N/R | N/R | N/R | N/R | |
|  | Dex 0.15mg/kg | 40 | N/R | N/R | N/R | N/R | N/R | 0 | 5 | 1 | N/R | N/R | N/R | N/R | N/R | N/R | N/R | N/R | N/R | |
|  | Pla | 40 | N/R | N/R | N/R | N/R | N/R | 3 | 3 | 1 | N/R | N/R | N/R | N/R | N/R | N/R | N/R | N/R | N/R | |
| Barros A, 2013 | Dex 4mg | 17 | N/R | N/R | N/R | N/R | N/R | N/R | N/R | N/R | N/R | N/R | N/R | N/R | N/R | N/R | N/R | N/R | N/R | |
|  | Pla | 17 | N/R | N/R | N/R | N/R | N/R | N/R | N/R | N/R | N/R | N/R | N/R | N/R | N/R | N/R | N/R | N/R | N/R | |
| Schietroma M, 2013 | Dex 8mg | 163 | N/R | N/R | N/R | N/R | N/R | N/R | N/R | N/R | N/R | N/R | N/R | N/R | N/R | N/R | N/R | N/R | N/R | |
|  | Pla | 165 | N/R | N/R | N/R | N/R | N/R | N/R | N/R | N/R | N/R | N/R | N/R | N/R | N/R | N/R | N/R | N/R | N/R | |
| Feroci F, 2011 | Dex 8mg | 51 | N/R | N/R | N/R | N/R | 0 | N/R | N/R | N/R | N/R | N/R | N/R | N/R | N/R | N/R | N/R | N/R | 1 | |
|  | Pla | 51 | N/R | N/R | N/R | N/R | 1 | N/R | N/R | N/R | N/R | N/R | N/R | N/R | N/R | N/R | N/R | N/R | 0 | |
| Eberhar LH, 1999 | Dro 5-7.5mg IV | 78 | N/R | N/R | N/R | N/R | N/R | N/R | N/R | N/R | N/R | N/R | N/R | N/R | N/R | N/R | N/R | N/R | N/R | |
|  | Mid 5-7.5mg IV | 72 | N/R | N/R | N/R | N/R | N/R | N/R | N/R | N/R | N/R | N/R | N/R | N/R | N/R | N/R | N/R | N/R | N/R | |
| Murmu A, 2015 | Ond 4mg | 63 | N/R | N/R | N/R | N/R | N/R | N/R | N/R | N/R | N/R | N/R | N/R | N/R | N/R | N/R | N/R | N/R | N/R | |
|  | Pla | 63 | N/R | N/R | N/R | N/R | N/R | N/R | N/R | N/R | N/R | N/R | N/R | N/R | N/R | N/R | N/R | N/R | N/R | |
| Song YK, 2013 | Pla | 41 | N/R | N/R | N/R | N/R | N/R | N/R | N/R | N/R | N/R | N/R | N/R | N/R | N/R | N/R | N/R | N/R | N/R | |
|  | Dex 10mg | 41 | N/R | N/R | N/R | N/R | N/R | N/R | N/R | N/R | N/R | N/R | N/R | N/R | N/R | N/R | N/R | N/R | N/R | |
|  | Ram 0.3mg IV | 41 | N/R | N/R | N/R | N/R | N/R | N/R | N/R | N/R | N/R | N/R | N/R | N/R | N/R | N/R | N/R | N/R | N/R | |
| Akin A, 2006 | Tro 5mg | 35 | N/R | N/R | N/R | N/R | N/R | N/R | N/R | N/R | N/R | N/R | N/R | N/R | N/R | N/R | N/R | N/R | N/R | |
|  | Tro 5mg + Pro 0.5mg/kg | 35 | N/R | N/R | N/R | N/R | N/R | N/R | N/R | N/R | N/R | N/R | N/R | N/R | N/R | N/R | N/R | N/R | N/R | |
|  | Pla | 35 | N/R | N/R | N/R | N/R | N/R | N/R | N/R | N/R | N/R | N/R | N/R | N/R | N/R | N/R | N/R | N/R | N/R | |
| Tarantino I, 2015 | Dex 8mg | 76 | N/R | N/R | N/R | N/R | N/R | N/R | N/R | N/R | 1 | 1 | 1 | 1 | 2 | N/R | N/R | N/R | N/R | |
|  | Pla | 76 | N/R | N/R | N/R | N/R | N/R | N/R | N/R | N/R | 1 | 0 | 0 | 0 | 0 | N/R | N/R | N/R | N/R | |
| Fujii Y, 2007 | Pla | 25 | N/R | N/R | N/R | N/R | N/R | N/R | N/R | N/R | N/R | N/R | N/R | N/R | N/R | N/R | N/R | N/R | N/R | |
|  | Dex 4mg | 25 | N/R | N/R | N/R | N/R | N/R | N/R | N/R | N/R | N/R | N/R | N/R | N/R | N/R | N/R | N/R | N/R | N/R | |
|  | Dex 8mg | 25 | N/R | N/R | N/R | N/R | N/R | N/R | N/R | N/R | N/R | N/R | N/R | N/R | N/R | N/R | N/R | N/R | N/R | |
| Papadima A, 2013 | Gra 3mg | 45 | 3 | | N/R | N/R | N/R | N/R | N/R | N/R | N/R | N/R | N/R | N/R | N/R | N/R | N/R | N/R | N/R | |
|  | Tro 5mg | 40 | 4 | | N/R | N/R | N/R | N/R | N/R | N/R | N/R | N/R | N/R | N/R | N/R | N/R | N/R | N/R | N/R | |
|  | Pla | 42 | 1 | | N/R | N/R | N/R | N/R | N/R | N/R | N/R | N/R | N/R | N/R | N/R | N/R | N/R | N/R | N/R | |
| Lee DC, 2011 | Pla | 65 | 29 | 13 | 11 | N/R | N/R | N/R | N/R | N/R | N/R | N/R | N/R | N/R | N/R | N/R | N/R | N/R | N/R | |
|  | Ram 0.3mg | 65 | 27 | 11 | 12 | N/R | N/R | N/R | N/R | N/R | N/R | N/R | N/R | N/R | N/R | N/R | N/R | N/R | N/R | |
| Tavlan A, 2006 | Dex | 60 | N/R | N/R | N/R | N/R | N/R | N/R | N/R | N/R | N/R | N/R | N/R | N/R | N/R | N/R | N/R | N/R | N/R | |
|  | Dex + Gin 0.5g oral | 60 | N/R | N/R | N/R | N/R | N/R | N/R | N/R | N/R | N/R | N/R | N/R | N/R | N/R | N/R | N/R | N/R | N/R | |
| Lee SY, 2002 | Pla | 41 | 9 | 2 | N/R | 3 | N/R | N/R | N/R | N/R | N/R | N/R | N/R | N/R | N/R | N/R | N/R | N/R | N/R | |
|  | Gra 20μg/kg | 36 | 9 | 2 | N/R | 5 | N/R | N/R | N/R | N/R | N/R | N/R | N/R | N/R | N/R | N/R | N/R | N/R | N/R | |
|  | Ram 4μg/kg | 36 | 13 | 5 | N/R | 2 | N/R | N/R | N/R | N/R | N/R | N/R | N/R | N/R | N/R | N/R | N/R | N/R | N/R | |
| Wang JJ, 1999 | Dex 10mg | 38 | N/R | N/R | N/R | N/R | N/R | N/R | N/R | N/R | N/R | N/R | N/R | N/R | N/R | N/R | N/R | N/R | N/R | |
|  | Dro 1.25mg IV | 40 | N/R | N/R | N/R | N/R | N/R | N/R | N/R | N/R | N/R | N/R | N/R | N/R | N/R | N/R | N/R | N/R | N/R | |
|  | Pla | 38 | N/R | N/R | N/R | N/R | N/R | N/R | N/R | N/R | N/R | N/R | N/R | N/R | N/R | N/R | N/R | N/R | N/R | |
| Zhang HW, 2016 | Dex 0.1mg/kg | 103 | N/R | N/R | N/R | N/R | N/R | N/R | N/R | N/R | N/R | N/R | N/R | N/R | N/R | N/R | N/R | N/R | N/R | |
|  | Pla | 130 | N/R | N/R | N/R | N/R | N/R | N/R | N/R | N/R | N/R | N/R | N/R | N/R | N/R | N/R | N/R | N/R | N/R | |
| Kim WJ, 2013 | Ram 0.3mg | 30 | N/R | N/R | N/R | N/R | N/R | N/R | N/R | N/R | N/R | N/R | N/R | N/R | N/R | N/R | N/R | N/R | N/R | |
|  | Mid 75μg/kg | 32 | N/R | N/R | N/R | N/R | N/R | N/R | N/R | N/R | N/R | N/R | N/R | N/R | N/R | N/R | N/R | N/R | N/R | |
|  | Ram 0.3mg + Mid 75μg/kg | 32 | N/R | N/R | N/R | N/R | N/R | N/R | N/R | N/R | N/R | N/R | N/R | N/R | N/R | N/R | N/R | N/R | N/R | |
| Worni M, 2008 | Pla | 35 | N/R | N/R | N/R | N/R | N/R | N/R | N/R | N/R | N/R | N/R | N/R | N/R | N/R | N/R | N/R | N/R | N/R | |
|  | Dex 8mg | 37 | N/R | N/R | N/R | N/R | N/R | N/R | N/R | N/R | N/R | N/R | N/R | N/R | N/R | N/R | N/R | N/R | N/R | |
| Wang JJ, 2000 | Dex10mg | 44 | N/R | N/R | N/R | N/R | N/R | N/R | N/R | N/R | N/R | N/R | N/R | N/R | N/R | N/R | N/R | N/R | N/R | |
|  | Dex 5mg | 43 | N/R | N/R | N/R | N/R | N/R | N/R | N/R | N/R | N/R | N/R | N/R | N/R | N/R | N/R | N/R | N/R | N/R | |
|  | Dex 2.5mg | 43 | N/R | N/R | N/R | N/R | N/R | N/R | N/R | N/R | N/R | N/R | N/R | N/R | N/R | N/R | N/R | N/R | N/R | |
|  | Dex 1.25mg | 44 | N/R | N/R | N/R | N/R | N/R | N/R | N/R | N/R | N/R | N/R | N/R | N/R | N/R | N/R | N/R | N/R | N/R | |
|  | Pla | 43 | N/R | N/R | N/R | N/R | N/R | N/R | N/R | N/R | N/R | N/R | N/R | N/R | N/R | N/R | N/R | N/R | N/R | |
| Fujii Y, 2001 | Pro 0.5mg.kg | 30 | 4 | 4 | N/R | N/R | N/R | N/R | N/R | N/R | N/R | N/R | N/R | N/R | N/R | N/R | N/R | N/R | N/R | |
|  | Dro 20μg/kg | 30 | 5 | 4 | N/R | N/R | N/R | N/R | N/R | N/R | N/R | N/R | N/R | N/R | N/R | N/R | N/R | N/R | N/R | |
|  | Met 0.2mg/kg | 30 | 4 | 4 | N/R | N/R | N/R | N/R | N/R | N/R | N/R | N/R | N/R | N/R | N/R | N/R | N/R | N/R | N/R | |
| Jokela R, 2002 | Ond 16mg | 60 | 38 | 27 | N/R | N/R | N/R | N/R | N/R | N/R | N/R | N/R | N/R | N/R | N/R | N/R | 11 | 8 | N/R | |
|  | Tro 5mg | 60 | 38 | 17 | N/R | N/R | N/R | N/R | N/R | N/R | N/R | N/R | N/R | N/R | N/R | N/R | 8 | 4 | N/R | |
|  | Met 10mg | 59 | 32 | 22 | N/R | N/R | N/R | N/R | N/R | N/R | N/R | N/R | N/R | N/R | N/R | N/R | 6 | 7 | N/R | |
| Lee MJ, 2015 | Pla | 36 | 1 | 0 | 1 | N/R | N/R | N/R | N/R | N/R | N/R | N/R | N/R | N/R | N/R | N/R | N/R | N/R | N/R | |
|  | Ram 0.3mg | 36 | 7 | 4 | 3 | N/R | N/R | N/R | N/R | N/R | N/R | N/R | N/R | N/R | N/R | N/R | N/R | N/R | N/R | |
|  | Ram 0.3mg + Dex 5mg | 36 | 3 | 3 | 3 | N/R | N/R | N/R | N/R | N/R | N/R | N/R | N/R | N/R | N/R | N/R | N/R | N/R | N/R | |
| Shilpa SNG, 2015 | Ond 8mg | 30 | 0 | N/R | N/R | N/R | N/R | N/R | N/R | N/R | N/R | N/R | N/R | N/R | N/R | 2 | N/R | N/R | N/R | |
|  | Clon 150μg | 30 | 2 | N/R | N/R | N/R | N/R | N/R | N/R | N/R | N/R | N/R | N/R | N/R | N/R | 0 | N/R | N/R | N/R | |

PONV: post-operative nausea and vomiting; IV: intravenous; Int: Intralipid; Ond: ondansetron; Pal: palonosetron; PCA: patient-controlled analgesia; IM: intramuscular; Pla: placebo; Gra: granisetron; Tro: tropisetron; Dex: dexamethasone; Pro: proprofol; Dia: diazepam; Ram: ramosetron; Dro: droperidol; Mid: midazolam; VAS: visual analogue pain score; TCI: target-controlled infusion; PAS: postanesthetic shivering; TCI: target-controlled infusion; SC: subcutaneous; Met: metoclopramide; Clon: clonidine; Gin: oral ginger; HTN: hypertension
