# Supplementary material for: Pharmacologic interventions for postoperative nausea and vomiting after thyroidectomy: A systematic review and network meta-analysis
Source: PLoS One. 2021 Jan 11;16(1):e0243865. doi: 10.1371/journal.pone.0243865 (PMC7799806; doi:10.1371/journal.pone.0243865)
Supplement: S3 Table — (DOCX) [file pone.0243865.s007.docx]

**Supplementary Table 3. Timing of pharmacological intervention**

| Study  (1^st^ author, year) | Country | Interventions | Timing of pharmacological intervention | Sample size | Outcome measurement for meta-analysis |
| --- | --- | --- | --- | --- | --- |
| Moon YE, 2012 | Republic of Korea | Ond 8mg bolus and 16mg in IV PCA | At the end of surgery | 50 | Incidence of PON, POV, PONV. Use of anti-emetics Severity of nausea Incidence of side-effects |
|  |  | Pal 0.075mg IV |  | 50 |  |
| Ewalenk P, 1996 | Belgium | Pro 0.1mg/kg/hr IV | At the end of surgery | 32 | Incidence of PON, POV, PONV. Use of anti-emetics Severity of PONV Sedation score |
|  |  | 10% Int 0.1mg/kg/hr IV |  | 32 |  |
| Metaxari M, 2011 | Greece | Pla 5mg IV | During induction | 50 | Incidence of PON, POV Severity of nausea |
|  |  | Gra 3mg IV |  | 50 |  |
|  |  | Ond 4mg IV |  | 51 |  |
|  |  | Tro 5mg IV |  | 52 |  |
| Zhou H, 2012 | China | Dex 8mg IV | Before induction | 50 | Incidence of PON, PONV. Use of anti-emetics. Complete response.  Postoperative pain Severity of PONV Postoperative pain intensity Adverse events, complications |
|  |  | Tro 5mg IV |  | 50 |  |
|  |  | Dex 8mg + Tro 5mg IV |  | 50 |  |
| Park JW, 2012 | Republic of Korea | Pal 0.075mg IV | Before induction | 41 | Incidence of PON, POV, PONV Severity of PONV Complete response |
|  |  | Pal 0.075mg + Dex 4mg IV |  | 43 |  |
| Jeon Y, 2010 | Republic of Korea | Ram 0.3mg IV | Immediately after induction | 60 | Incidence of PON, POV Severity of PONV Use of rescue antiemetics Occurrence of adverse events |
|  |  | Dex 8mg IV |  | 60 |  |
|  |  | Ram 0.3mg + Dex 8mg IV |  | 60 |  |
| Doksrod S, 2012 | Norway | Dex 0.3mg/kg IV | Immediately after induction | 40 | Incidence of PONV Severity of PONV Use of rescue antiemetics or analgesics Occurrence of side effects |
|  |  | Dex 0.15mg/kg IV |  | 40 |  |
|  |  | Pla |  | 40 |  |
| Barros A, 2013 | Portugal | Dex 4mg IV | Immediately after induction | 17 | Severity of PON, POV Use of the PCA pump Pain intensity Sedation and shivering scores Use of rescue antiemetics or analgesics |
|  |  | Pla |  | 17 |  |
| Schietroma M, 2013 | Italy | Dex 8mg IV | Before induction | 163 | Incidence of recurrent laryngeal nerve palsy  Use of rescue antiemetics or analgesics |
|  |  | Pla |  | 165 |  |
| Eberhar LH, 1999 | Germany | Dro 5-7.5mg IV *5mg: body weight<70kg,  7.5mg: body weight≥70kg | During induction | 78 | Post-operative mood and well-being Incidence of PON , POV Impact of PONV on post-operative mood and well-being Use of rescue antiemetics or analgesics |
|  |  | Mid 5-7.5mg IV *5mg: body weight<70kg,  7.5mg: body weight≥70kg |  | 72 |  |
| Song YK, 2013 | Republic of Korea | Pla | Immediately after induction | 41 | Incidence of PON, POV and PONV Severity of PONV Use of rescue antiemetics Severity of PAS Post-operative pain (VAS) |
|  |  | Dex 10mg IV |  | 41 |  |
|  |  | Ram 0.3mg IV |  | 41 |  |
| Akin A, 2006 | Turkey | Tro 5mg IV | Immediately after induction | 35 | Post-operative pain (VAS) Incidence of PON, POV Use of rescue antiemetics Complete response |
|  |  | Tro 5mg + Pro 0.5mg/kg IV |  | 35 |  |
|  |  | Pla |  | 35 |  |
| Tarantino I, 2015 | Germany | Dex 8mg IV | Before induction | 76 | Incidence of PON, POV Severity of PONV Severity of pain, length of stay Severity of adverse events |
|  |  | Pla |  | 76 |  |
| Fujii Y, 2007 | Japan | Pla | At the end of surgery | 25 | Incidence of PON, POV Severity of nausea Post-operative pain |
|  |  | Dex 4mg IV |  | 25 |  |
|  |  | Dex 8mg IV |  | 25 |  |
| Papadima A, 2013 | Greece | Gra 3mg IV | Before induction | 45 | Post-operative pain (VAS) Incidence of PON, POV Severity of PON, POVV Use of rescue antiemetics Side effects |
|  |  | Tro 5mg IV |  | 40 |  |
|  |  | Pla |  | 42 |  |
| Lee DC, 2011 | Republic of Korea | Pla | At the end of surgery | 65 | Incidence of PON, POV Severity of PONV Use of rescue anti-emetics and analgesics Complete response Pain score Side effects of antiemetics |
|  |  | Ram 0.3mg IV |  | 65 |  |
| Tavlan A, 2006 | Turkey | Dex | Before induction | 60 | Incidence of PON, POV Severity of PON, POV Use of rescue analgesics, antiemetics |
|  |  | Dex + Gin 0.5g oral |  | 60 |  |
| Lee SY, 2002 | Republic of Korea | Pla | Before induction | 41 | Incidence of PON, POV, PONV Severity of PONV Adverse events Use of rescue antiemetics |
|  |  | Gra 20μg/kg IV |  | 36 |  |
|  |  | Ram 4μg/kg IV |  | 36 |  |
| Wang JJ, 1999 | Taiwan | Dex 10mg IV | Before induction | 38 | Incidence of PON, PONV Severity of PON Post-operative pain (VAS) Occurrence of sore throat, restlessness |
|  |  | Dro 1.25mg IV |  | 40 |  |
|  |  | P |  | 38 |  |
| Zhang HW, 2016 | China | Dex 0.1mg/kg IV | During induction | 103 | Incidence of PON, POV,  Use of rescue anti-emetics Post-operative pain (VAS) Blood glucose level |
|  |  | Pla |  | 130 |  |
| Kim WJ, 2013 | Republic of Korea | Ram 0.3mg IV | Before induction | 30 | Incidence of POV Severity of PON Post-operative pain (VAS) Use of rescue anti-emetics |
|  |  | Mid 75μg/kg IV |  | 32 |  |
|  |  | Ram 0.3mg + Mid 75μg/kg IV |  | 32 |  |
| Worni M, 2008 | Switzerland | Pla | Before induction | 35 | Incidence of PON, POV and PONV Severity of PON Post-operative pain (VAS) Voice function Severity of use of rescue anti-emetics, analgesics |
|  |  | Dex 8mg IV |  | 37 |  |
| Wang JJ, 2000 | Taiwan | Dex10mg | Immediately after induction | 44 | Incidence of PON, POV Severity of PON, POV Use of rescue antiemetics, analgesics Complete response Post-operative pain (VAS) Side effects |
|  |  | Dex 5mg |  | 43 |  |
|  |  | Dex 2.5mg |  | 43 |  |
|  |  | Dex 1.25mg |  | 44 |  |
|  |  | Pla |  | 43 |  |
| Fujii Y, 2001 | Japan | Pro 0.5mg.kg IV | At the end of surgery | 30 | Incidence of PON, POV, PONV Severity of PON Sedation score Use of rescue antiemetics |
|  |  | Dro 20μg/kg IV |  | 30 |  |
|  |  | Met 0.2mg/kg IV |  | 30 |  |
| Jokela R, 2002 | Finland | Ond 16mg IV | Before induction | 60 | Incidence of PON, PONV Severity of PONV Use of rescue antiemetics, analgesics Post-operative pain (VAS) Incidence of adverse events |
|  |  | Tro 5mg IV |  | 60 |  |
|  |  | Met 10mg IV |  | 59 |  |
| Lee MJ, 2015 | Republic of Korea | Pla | Immediately after induction | 36 | Incidence of PON, POV Severity of PON, POV Post-operative pain (VAS) Incidence of adverse events Use of rescue antiemetics, analgesics |
|  |  | Ram 0.3mg |  | 36 |  |
|  |  | Ram 0.3mg + Dex 5mg |  | 36 |  |

PONV: post-operative nausea and vomiting; IV: intravenous; Ond: ondansetron; Pal: palonosetron; PCA: patient-controlled analgesia; IM: intramuscular; Pla: placebo; Gra: granisetron; Tro: tropisetron; Dex: dexamethasone; Pro: proprofol; Dia: diazepam; Ram: ramosetron; Dro: droperidol; Mid: midazolam; VAS: visual analogue pain score; TCI: target-controlled infusion; PAS: post-anesthetic shivering; TCI: target-controlled infusion; SC: subcutaneous; Met: metoclopramide; Gin: oral ginger
