# Supplementary figures and images for: Pharmacologic interventions for postoperative nausea and vomiting after thyroidectomy: A systematic review and network meta-analysis
Source: PLoS One. 2021 Jan 11;16(1):e0243865. doi: 10.1371/journal.pone.0243865 (PMC7799806; doi:10.1371/journal.pone.0243865)

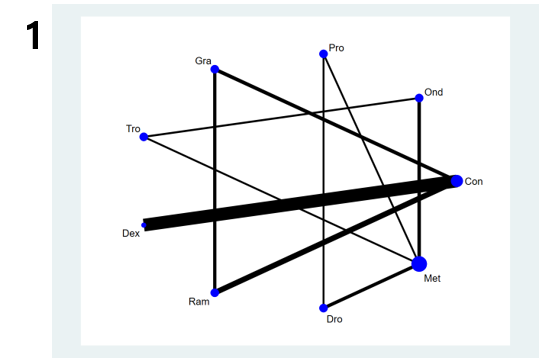

Supplement: S1 Fig — (TIF) [file pone.0243865.s008.tif]

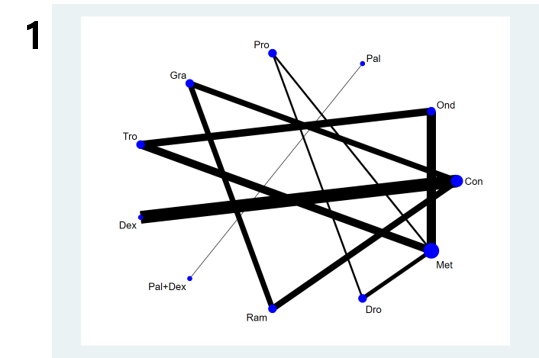

Supplement: S2 Fig — (TIF) [file pone.0243865.s009.tif]

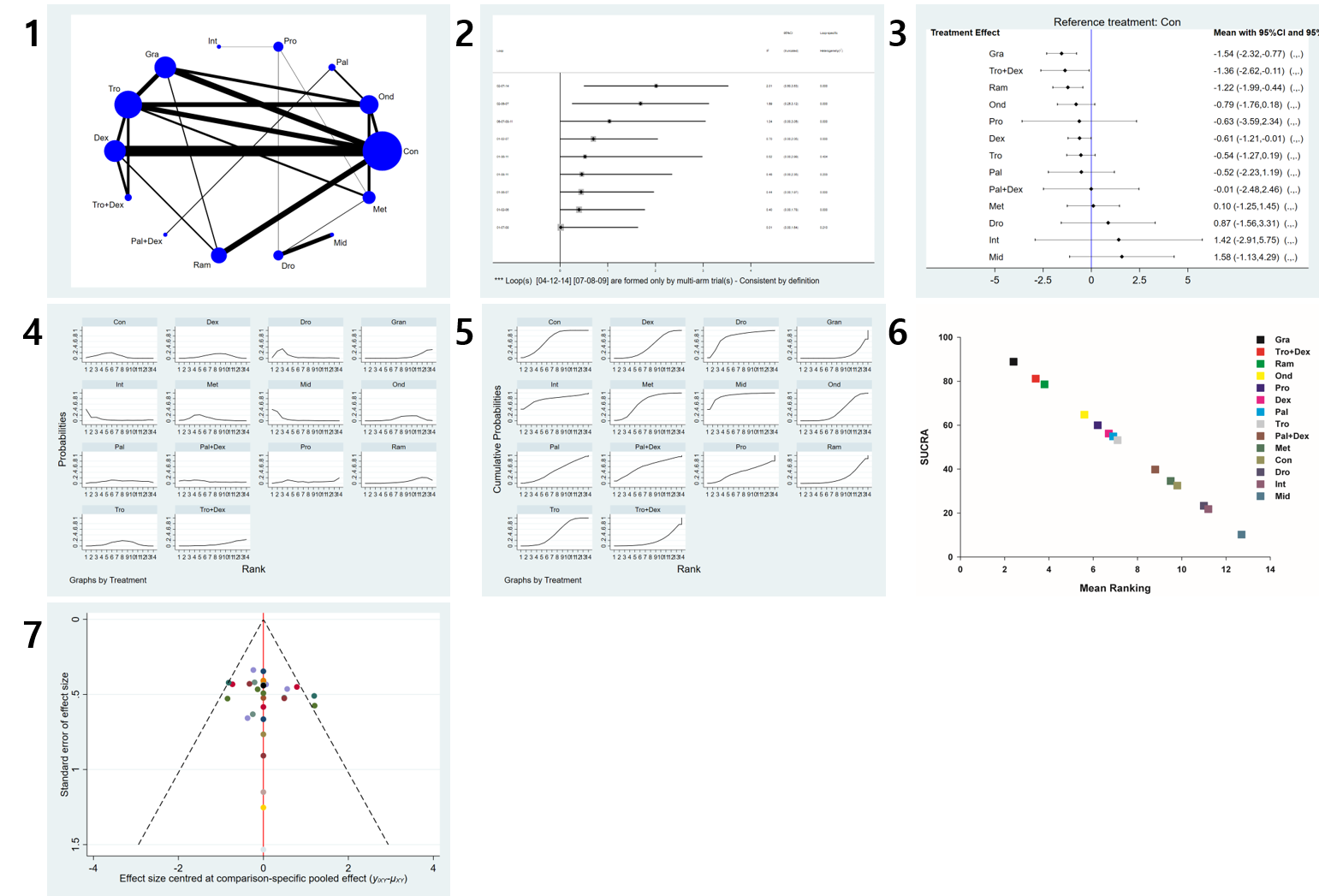

Supplement: S3 Fig — (TIF) [file pone.0243865.s010.tif]

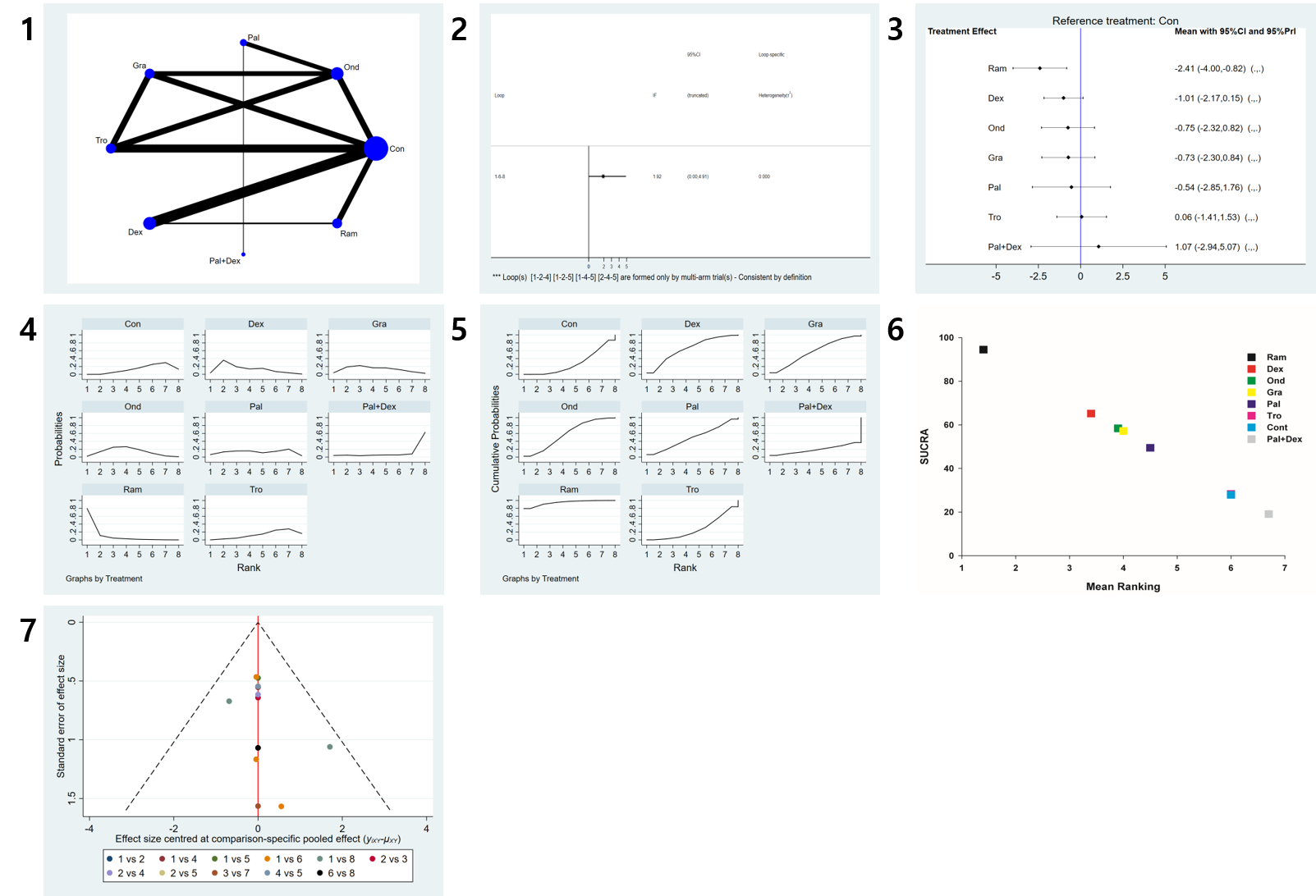

Supplement: S4 Fig — (TIF) [file pone.0243865.s011.tif]

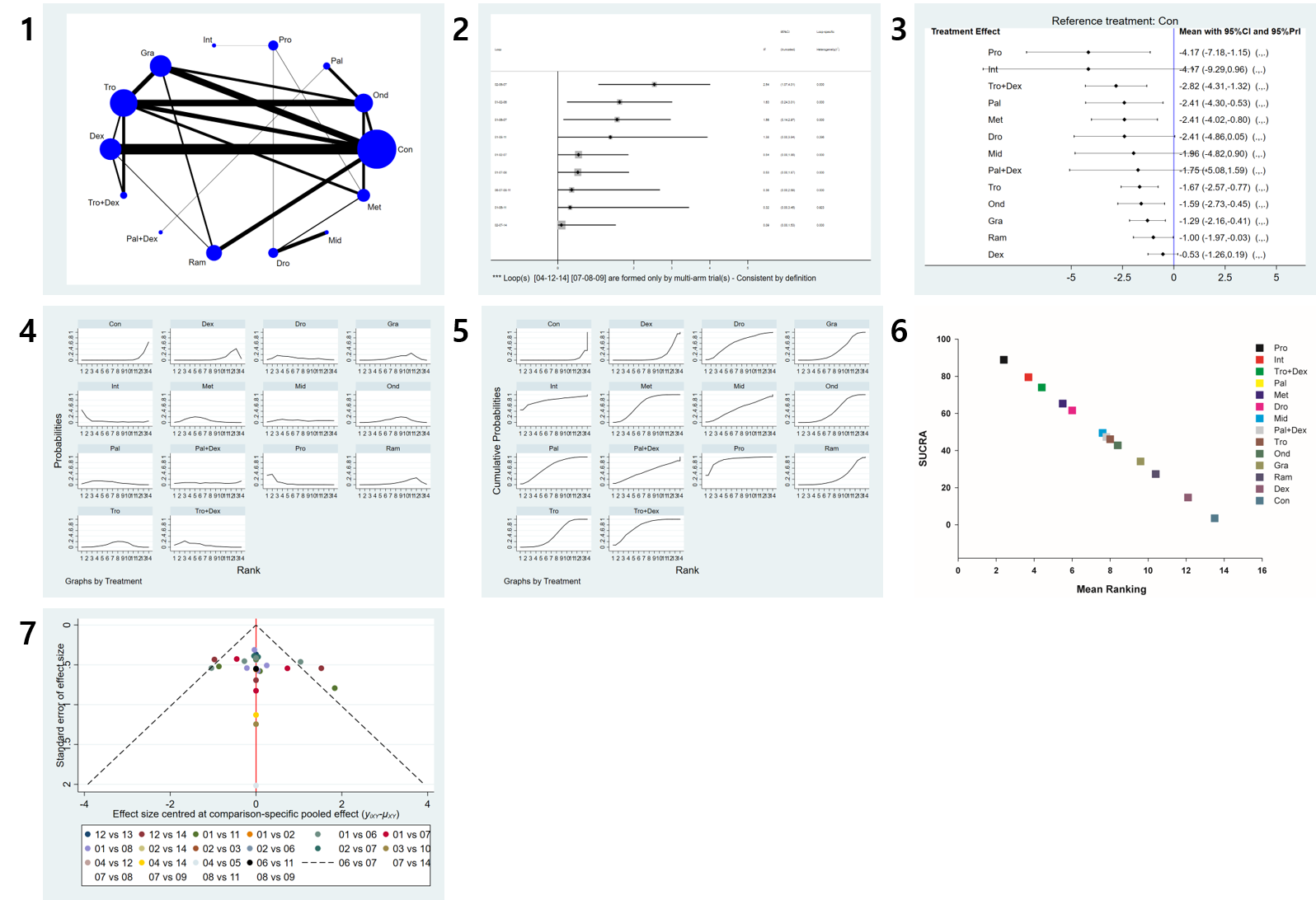

Supplement: S5 Fig — (TIF) [file pone.0243865.s012.tif]

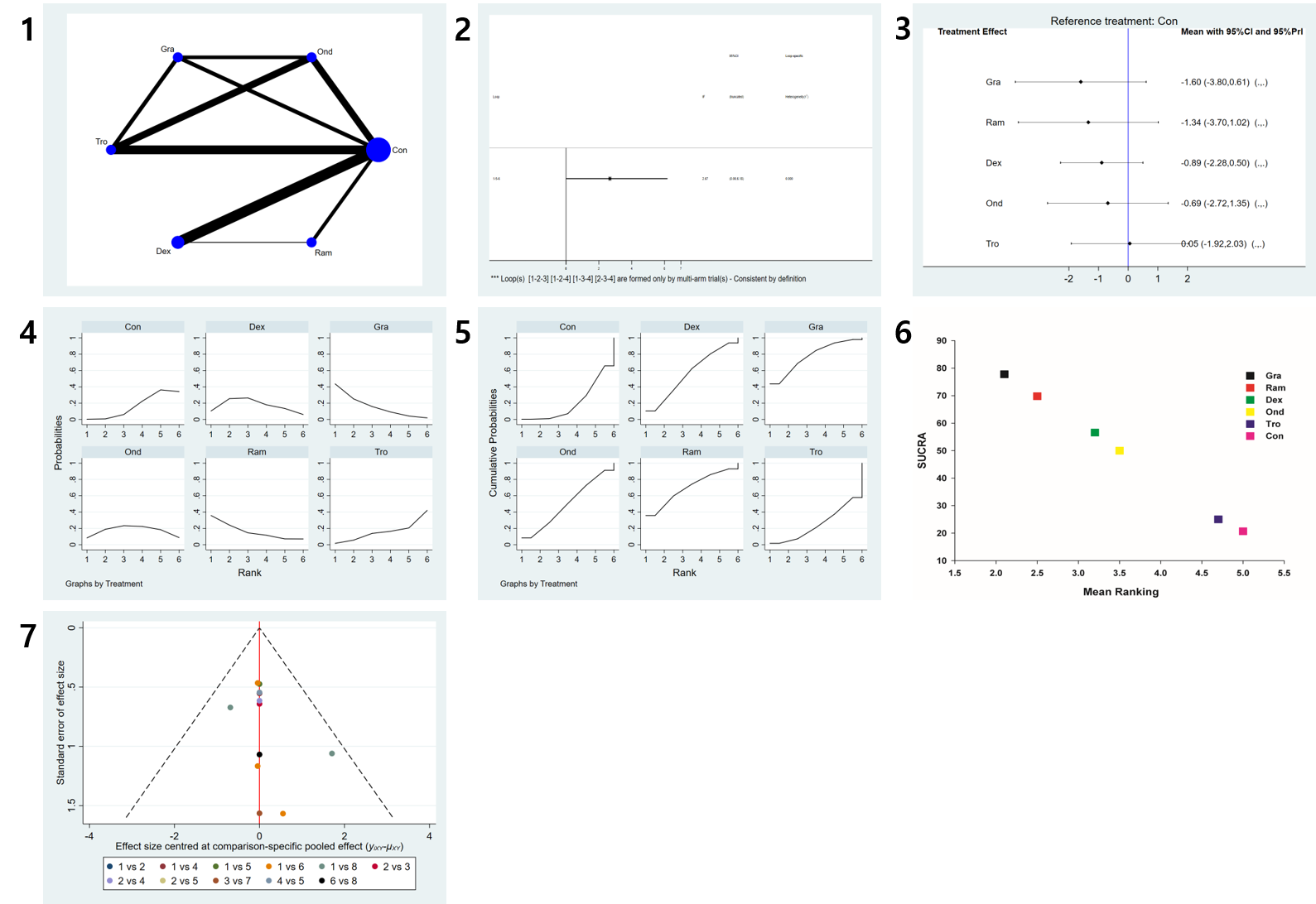

Supplement: S6 Fig — (TIF) [file pone.0243865.s013.tif]

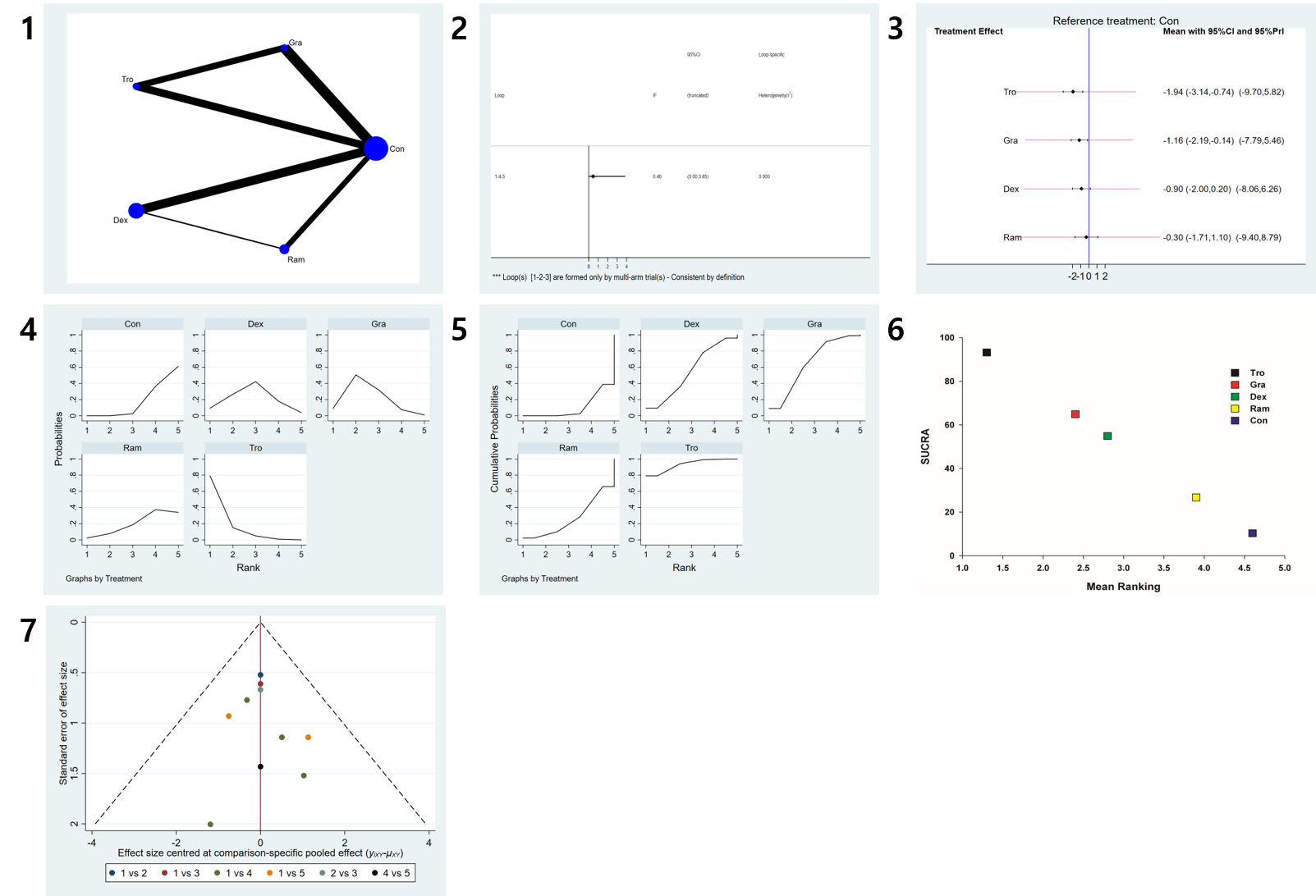

Supplement: S7 Fig — (TIF) [file pone.0243865.s014.tif]

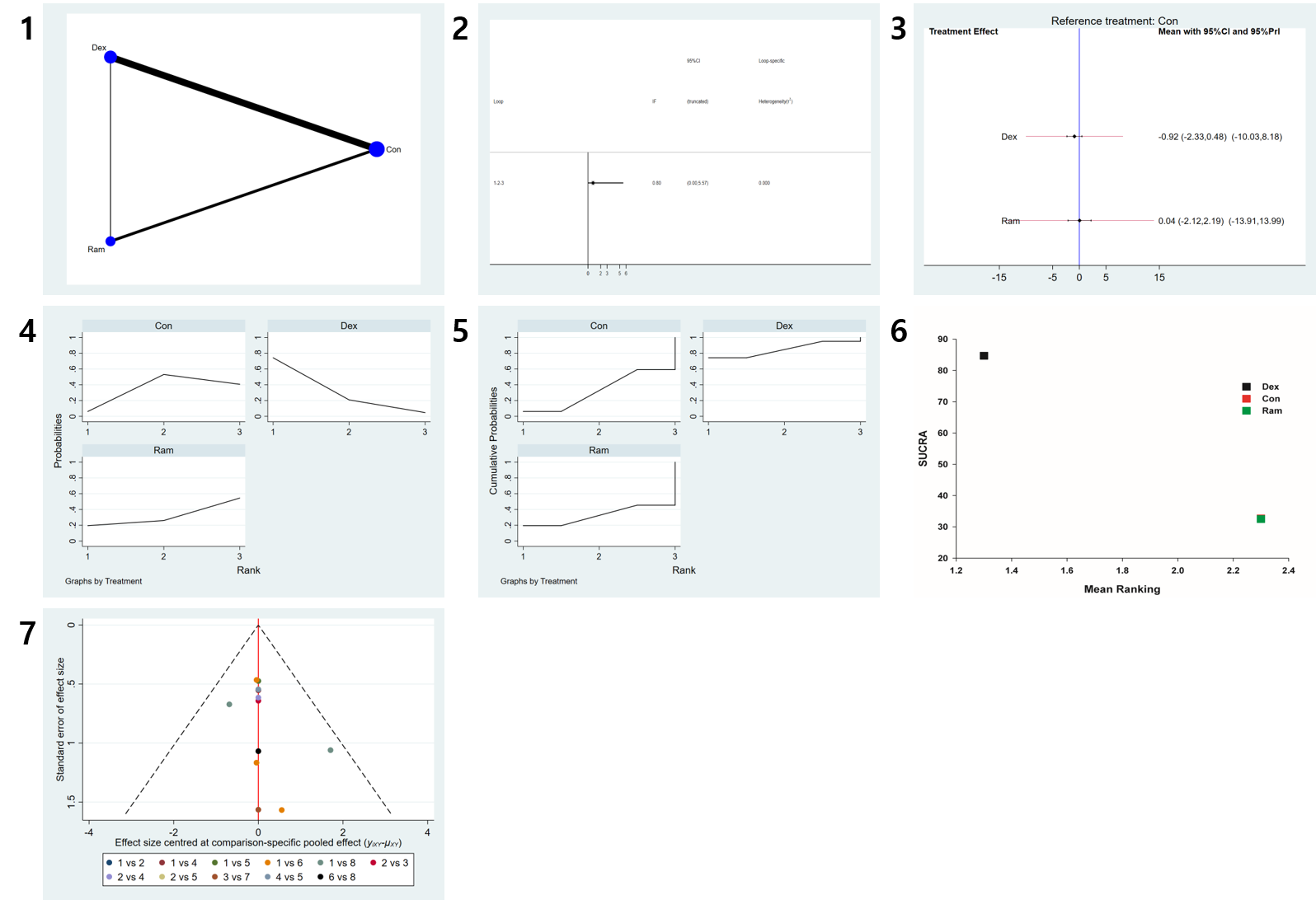

Supplement: S8 Fig — (TIF) [file pone.0243865.s015.tif]

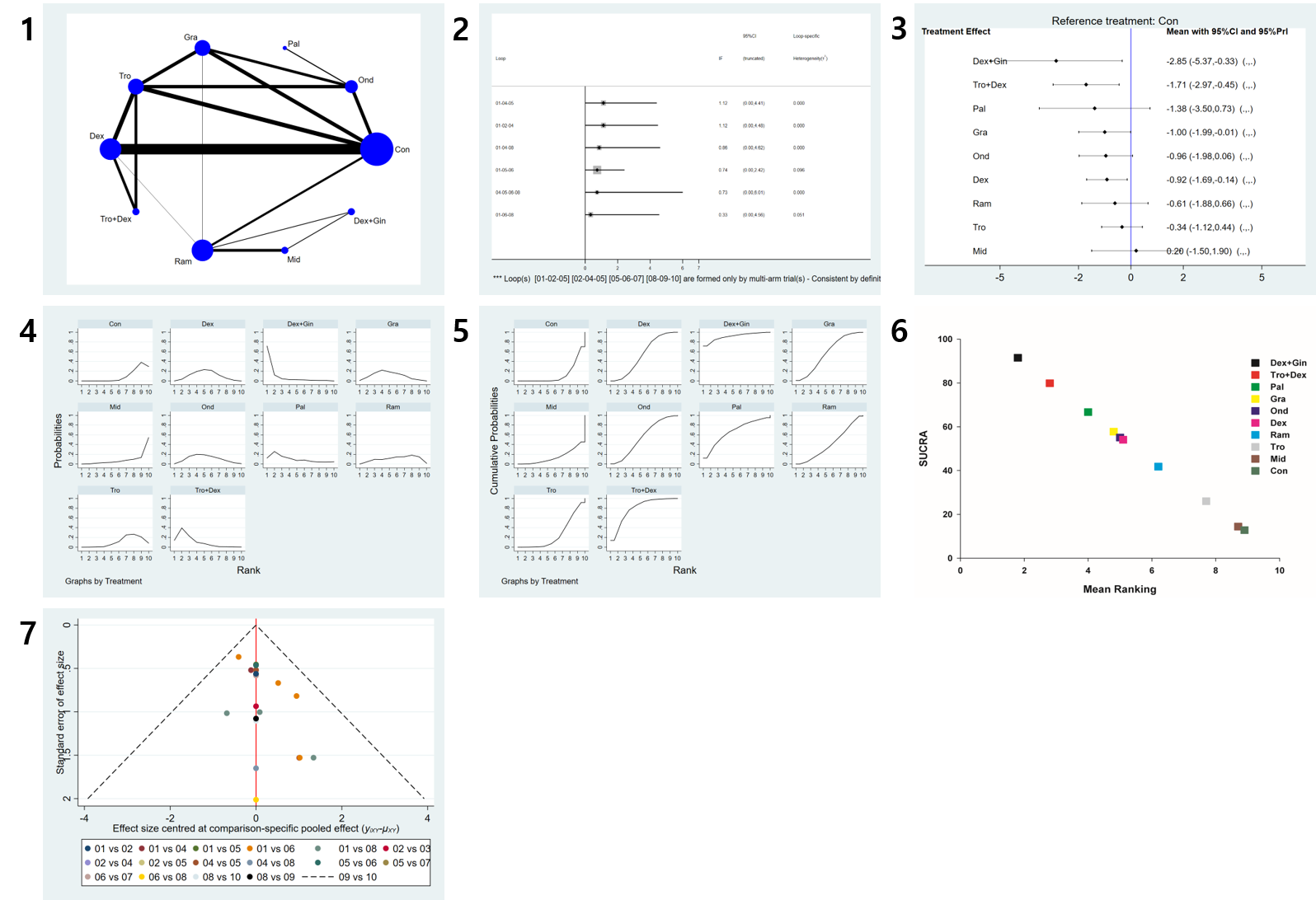

Supplement: S9 Fig — (TIF) [file pone.0243865.s016.tif]

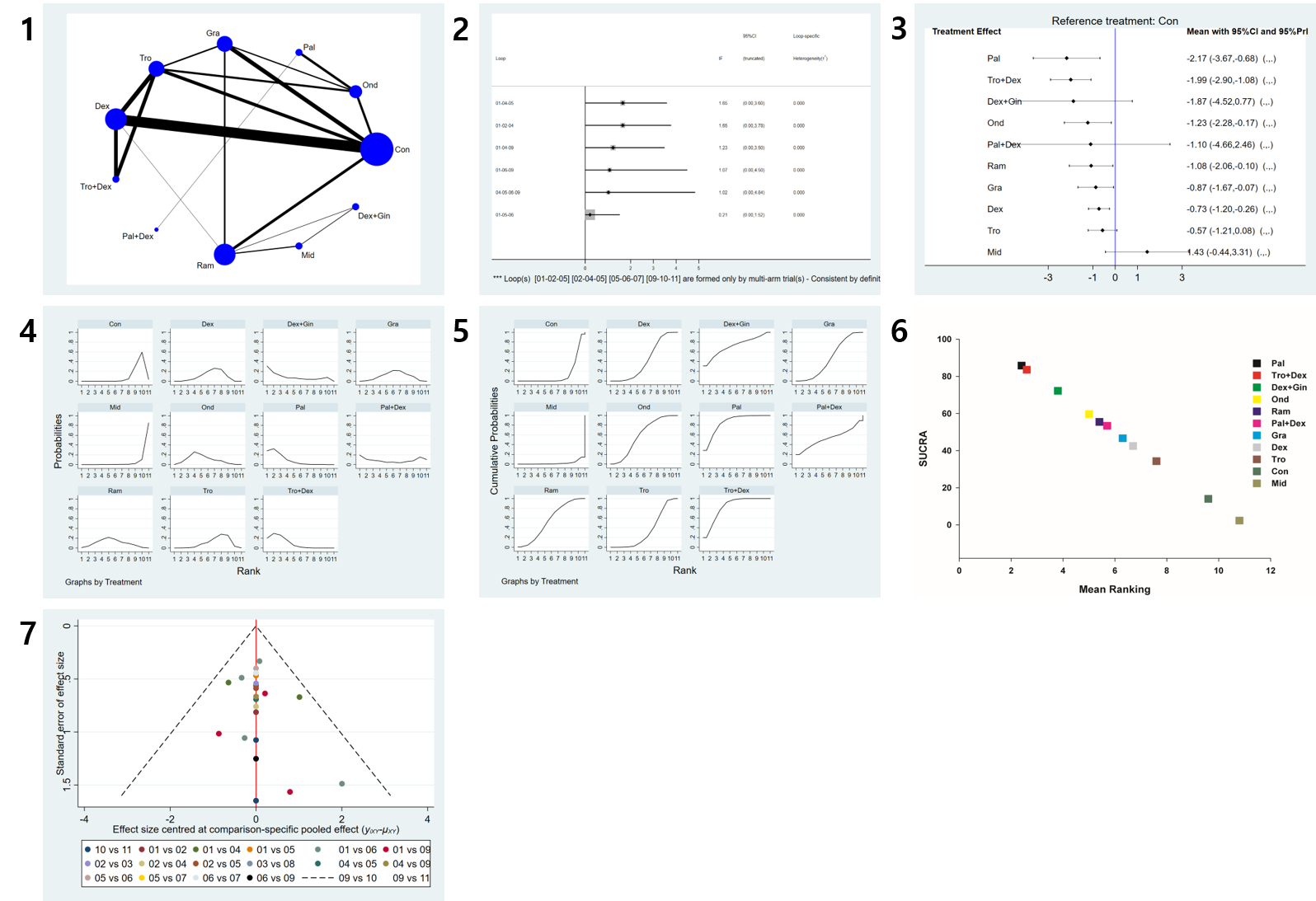

Supplement: S10 Fig — (TIF) [file pone.0243865.s017.tif]

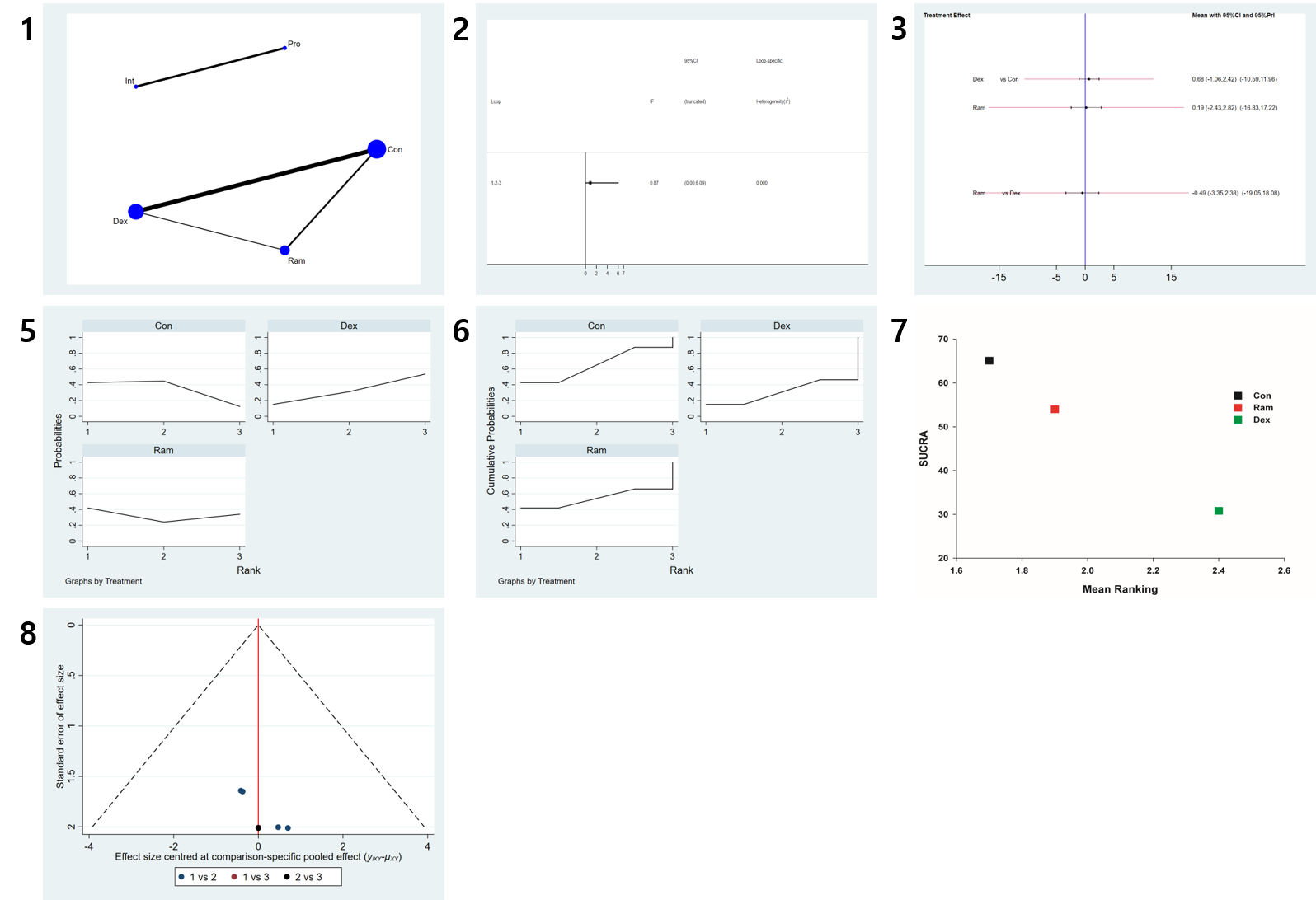

Supplement: S11 Fig — (TIF) [file pone.0243865.s018.tif]

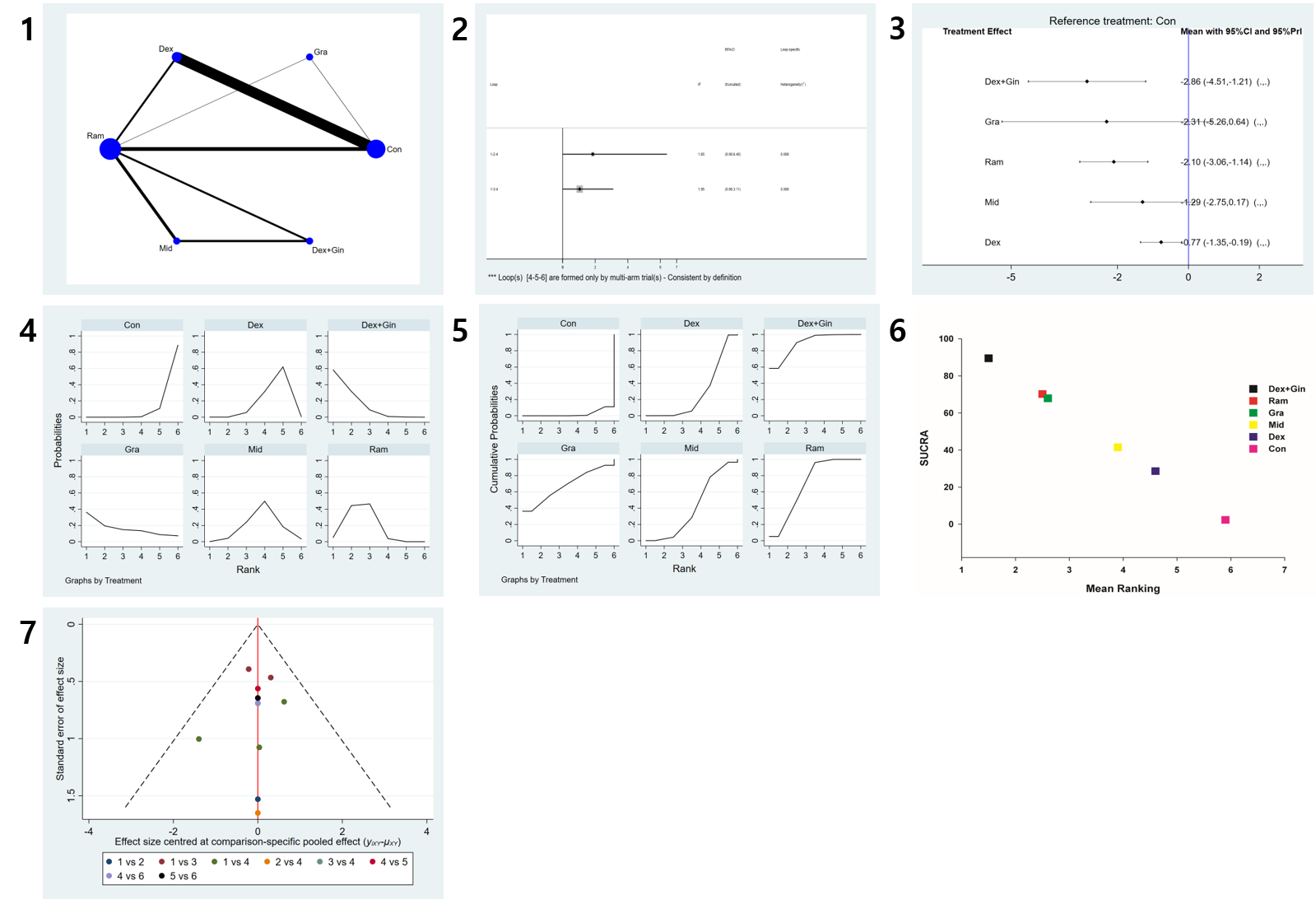

Supplement: S12 Fig — (TIF) [file pone.0243865.s019.tif]

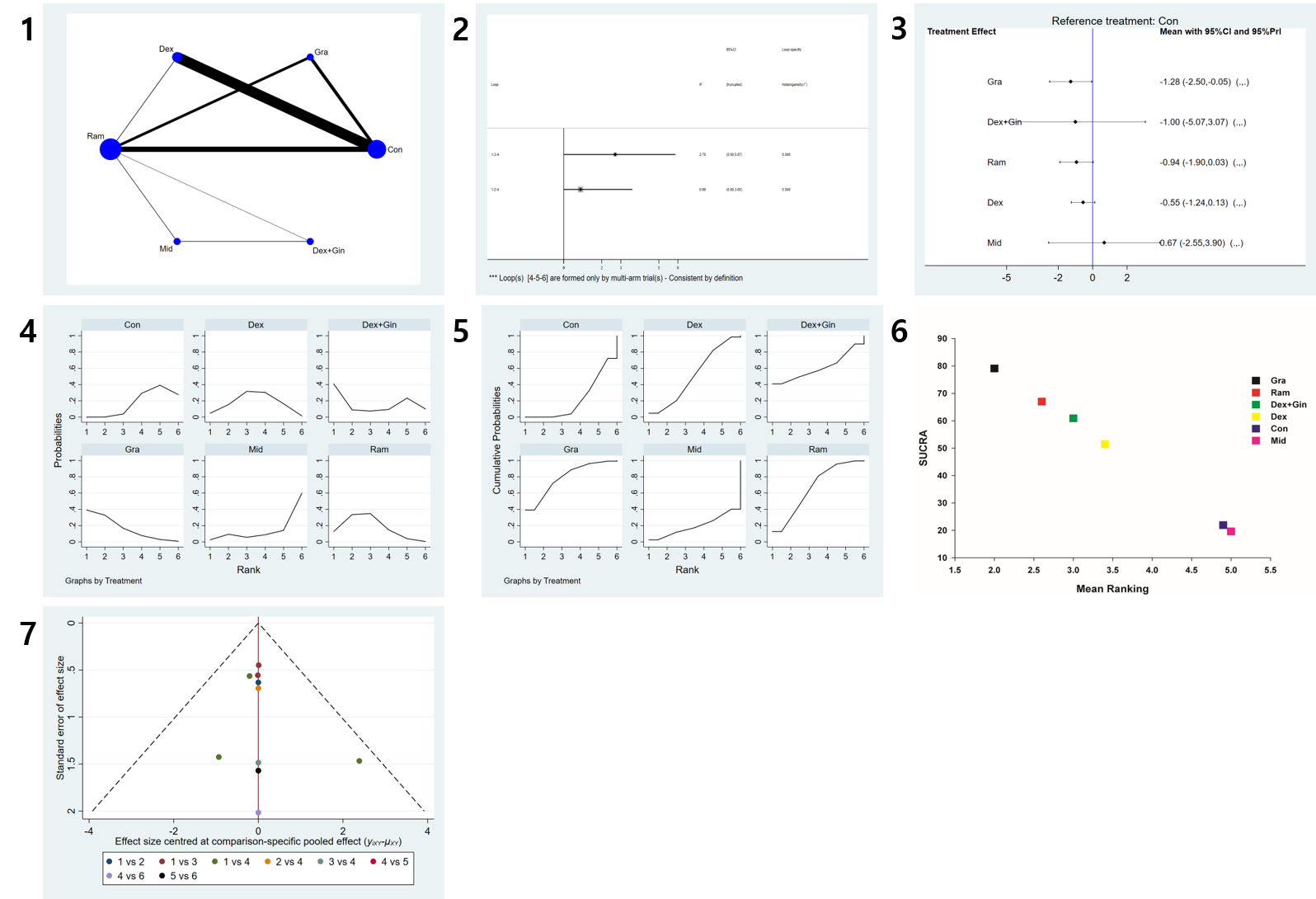

Supplement: S13 Fig — (TIF) [file pone.0243865.s020.tif]

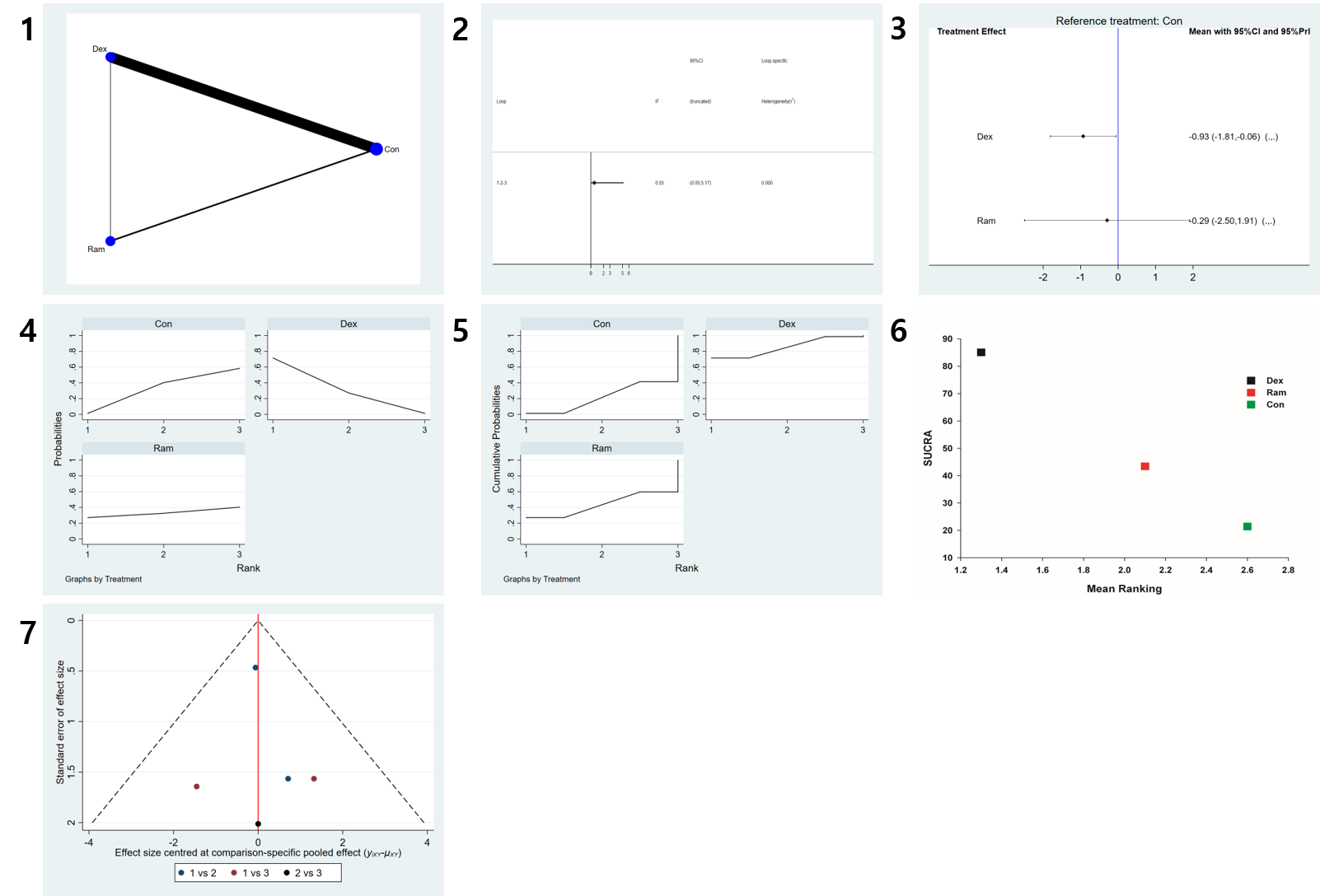

Supplement: S14 Fig — (TIF) [file pone.0243865.s021.tif]
