# Supplementary material for: Pharmacologic interventions for postoperative nausea and vomiting after thyroidectomy: A systematic review and network meta-analysis
Source: PLoS One. 2021 Jan 11;16(1):e0243865. doi: 10.1371/journal.pone.0243865 (PMC7799806; doi:10.1371/journal.pone.0243865)
Supplement: Attachment — Submitted filename: Answer letter.docx [file pone.0243865.s023.docx]

**November 25, 2020**

**Dear Dr Ivan D. Florez, Academic Editor of ‘*Plos One*’,**

**Re: Manuscript number PONE-D-20-20400R1, “Pharmacologic interventions for postoperative nausea and vomiting after thyroidectomy: a systematic review and network meta-analysis”**

We thank sincerely the academic editor and reviewers of the ‘*Plos One*’ for taking their precious time to review our paper. According to your comments and suggestions, we sincerely and earnestly tried to response for your comments. We want to express my heartfelt gratitude for your comments once more.

We have made some corrections in the manuscript after going over your comments. We highlighted the modification made to the original document by using red colored text. The changes are summarized below:

**Academic editor’s comment**

**Thanks for submitting your revised version, which has been reviewed by one of the initial reviewers and myself as Editor.
In response to our comments you have provided responses and edits to your manuscript. There are still some points that need to be addressed:

- Thanks for adding a more detailed explanation of the PrI. However, this information requires an appropriate citation.**

**Our response**: Thank you for editor’s suggestions for the betterment of the manuscript. According to editor’s suggestion, we cited appropriate citation**(line 237 on page 12 in the Methods section)**

**- Thanks for considering the League tables. Please add footnotes that may explain the readers how to read and interpret them. You may want to use the following paper as an example: https://www.thelancet.com/journals/lancet/article/PIIS0140-6736(17)32802-7/fulltext**

**Our response**: Thank you for editor’s suggestions for the betterment of the manuscript. According to editor’s suggestion, we added the foot notes. **(Table 3, 4, 5, 6, 7)**

We hope the revised manuscript will better meet the requirements of the ‘*Plos One*’ for publication. Again, we are most grateful for the constructive review by you and members of Editorial Board of the ‘*Plos One*’.

Sincerely yours,

Hyun Kang, M. D., Ph.D., M.P.H.

Professor

Department of Anaesthesiology and Pain Medicine

Chung-Ang University College of Medicine

224-1 Heukseok-dong, Dongjak-gu

Seoul, 156-755, Korea

Tel: +82-2-6299-2571, 2579, 2586

Mobile:+82-10-8761-4426

Fax: 82-2-6299-2585

E-mail: [roman00@naver.com](mailto:roman00@naver.com)
